# Generation of antagonistic biparatopic anti-CD30 antibody from an agonistic antibody by precise epitope determination and utilization of structural characteristics of CD30 molecule

**DOI:** 10.1093/abt/tbaf002

**Published:** 2025-01-14

**Authors:** Hiroki Akiba, Tomoko Ise, Reiko Satoh, Yasuhiro Abe, Kouhei Tsumoto, Hiroaki Ohno, Haruhiko Kamada, Satoshi Nagata

**Affiliations:** Graduate School of Pharmaceutical Sciences, Kyoto University, Sakyo-ku, Kyoto 606-8501, Japan; Laboratory of Advanced Biopharmaceuticals, Center for Drug Design Research, National Institutes of Biomedical Innovation, Health and Nutrition, Ibaraki, Osaka 567-0085, Japan; Laboratory of Antibody Design, Center for Drug Design Research, National Institutes of Biomedical Innovation, Health and Nutrition, Ibaraki, Osaka 567-0085, Japan; Laboratory of Advanced Biopharmaceuticals, Center for Drug Design Research, National Institutes of Biomedical Innovation, Health and Nutrition, Ibaraki, Osaka 567-0085, Japan; Division of Drugs, National Institute of Health Sciences, Kawasaki-ku, Kawasaki, Kanagawa 210-9501, Japan; School of Engineering, The University of Tokyo, Bunkyo-ku, Tokyo 113-8656, Japan; The Institute of Medical Science, The University of Tokyo, Minato-ku, Tokyo 108-8639, Japan; Graduate School of Pharmaceutical Sciences, Kyoto University, Sakyo-ku, Kyoto 606-8501, Japan; Laboratory of Advanced Biopharmaceuticals, Center for Drug Design Research, National Institutes of Biomedical Innovation, Health and Nutrition, Ibaraki, Osaka 567-0085, Japan; Graduate School of Pharmaceutical Sciences, Kyoto University, Sakyo-ku, Kyoto 606-8501, Japan; Laboratory of Advanced Biopharmaceuticals, Center for Drug Design Research, National Institutes of Biomedical Innovation, Health and Nutrition, Ibaraki, Osaka 567-0085, Japan; Laboratory of Antibody Design, Center for Drug Design Research, National Institutes of Biomedical Innovation, Health and Nutrition, Ibaraki, Osaka 567-0085, Japan

**Keywords:** CD30 receptor, TNFRSF8, biparatopic antibody, biepitopic antibody, epitope targeting, bispecific antibody

## Abstract

**Background:**

CD30 is a member of the tumor necrosis factor receptor superfamily. Recently, blocking CD30-dependent intracellular signaling has emerged as potential strategy for immunological regulation. Development of antibody-based CD30 antagonists is therefore of significant interest. However, a key challenge is that the bivalent form of natural antibody can crosslink CD30 molecules, leading to signal transduction even in the absence of specific ligand, CD153. Biparatopic antibodies (BpAbs) offer a solution, using two different variable fragments (Fvs) to bind distinct epitopes on a single antigen molecule. BpAbs format is an attractive alternative of natural antibody by potentially avoiding unwanted crosslinking and signaling induction.

**Methods:**

We systematically characterized 36 BpAbs, each designed with pairs of Fvs binding to nine distinct epitopes across the CD30 extracellular domain. We first identified the precise epitope sites of the nine antibodies by assessing the binding to multiple orthologous CD30 proteins and mutants. We then produced the 36 BpAbs and analyzed their biological activities and binding modes.

**Results:**

Among 36 BpAbs, we identified both potent ligand-independent agonists and ligand-blocking antagonists, with many displayed reduced signal activation, including 1:1-binding antagonists derived from AC10, a strong agonist developed for lymphoma therapy. Epitope dependency in reduced signaling activity was observed and associated with the flexible nature of CD30 protein.

**Conclusions:**

We successfully developed antagonistic BpAbs against CD30 by controlling the stoichiometry of antibody–antigen binding mode. This study elucidated the mechanism of signaling induction, informing the design strategies of the development of biparatopic antibodies.

## Teaser

Statement of Significance: Thirty-six bivalent biparatopic antibodies targeting pairs of nine distinct topographical epitopes distributed over CD30 were developed. These BpAbs exhibited a range of signaling activities from agonism to antagonism, with many displaying reduced signal activation. Notably, 1:1-binding antagonists were developed with no signal induction. The mechanism was elucidated by epitope information.

## Introduction

Human CD30 (UniProt ID: P28908) has been pursued as a target for antibody therapy for lymphoma. CD30 was initially discovered as an overexpressed cell surface molecule in Hodgkin’s lymphoma and anaplastic large cell lymphoma. It was later identified as being encoded by member 8 of tumor necrosis factor receptor super family gene (TNFRSF8), with its expression primarily restricted in activated lymphocytes under normal conditions [1–4]. Consequently, many anti-CD30 monoclonal antibodies (mAbs) were evaluated for lymphoma therapy. The moderate potency of most of the mAbs hindered progress until the approval of brentuximab vedotin, an antibody-drug conjugate, successfully introduced in the market [5]. Besides the potent anti-mitotic activity of monomethyl auristatin E, brentuximab vedotin utilizes an antibody known as AC10, originally developed as an agonist as naked antibody that failed to demonstrate significant advantages [6, 7]. Antagonists targeting CD30 have not yet been developed although CD30-mediated signaling including NF-κB pathway whose aberrant activation may contribute on a variety of human diseases, including inflammatory and autoimmune diseases [8].

**Figure 1:**
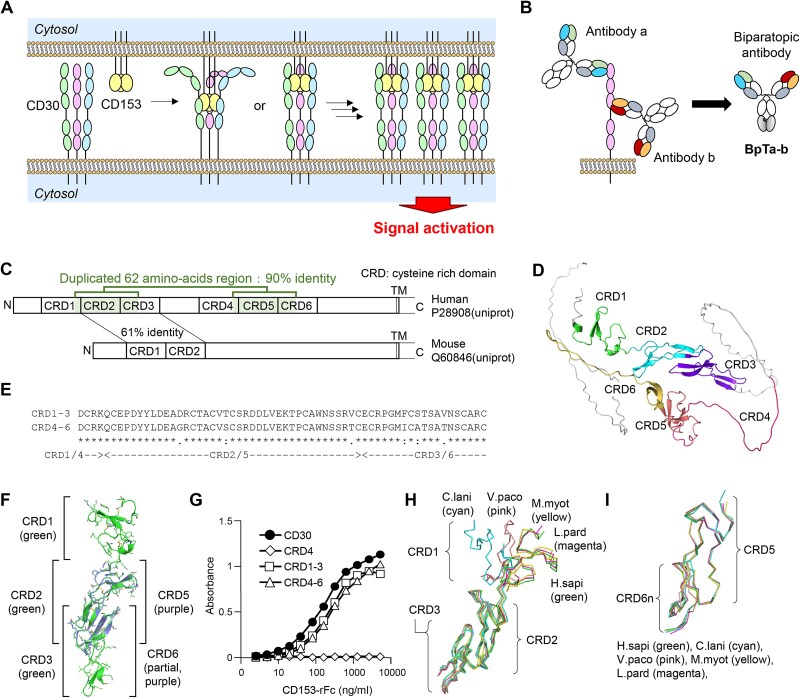
CD30 and biparatopic antibodies (BpAbs). (A) Activation mechanism of CD30. CD30 has two regions binding the specific trimeric ligand (CD153). With the association of CD153, CD30 is expected to form clusters on the cell membrane, and downstream signal cascade is activated. (B) Bivalent BpAbs. In this study, BpAbs are named as BpTa-b after two originating antibodies (antibody a and antibody b). (C) Schematic illustration of human CD30 with duplicate and ligand-binding regions. (D) Presence of disordered regions. In addition to the region between CRD3 and CRD4, CRD4 (magenta) and C-terminal half of CRD6 are predicted to be disordered in AlphaFold2. (E) Similarity of CRD2-3 and CRD5-6. (F) Superimposed AlphaFold-predicted structures of CRD1-3 and CRD5-6n from human CD30. (G) Binding of CD153 both to CRD1-3 and CRD4-6 recombinant proteins. (H) Superimposed AlphaFold-predicted structures of CRD5-6n from human and five orthologs. (I) Superimposed AlphaFold-predicted structures of CRD1-3 from human and five orthologs.

Under natural conditions, activation of CD30 occurs through binding with its trimeric ligand, CD153 (or CD30L). Members of the TNFRSF, such as CD30, contain variable numbers of cysteine-rich domains (CRDs) in their extracellular regions, which form their ligand binding sites. CRDs, commonly observed in > 20 TNFRSF members, share a common fundamental higher order structure [9, 10]. The ligand molecules of the tumor necrosis factor superfamily, including CD153, are trimeric and induce clustering of corresponding TNFRSF on the cell membrane through their interaction (Fig. 1A) [1, 11]. The reassembly of TNFRSF into cluster configurations involves conformational changes in the intracellular regions at a macromolecular level, and downstream signaling molecules such as TNFR-associated factors are recruited to activate NF-κB pathway [8].

It is well established that TNFRSF signaling plays crucial and diverse immunomodulatory roles in various pathological conditions, including a range of immune pathologies, cancers, and infectious diseases [12–14]. Given the frequent association of the CD30-CD153 axis with immunological disorders [15–22], antagonists are anticipated as drug candidates for immunological regulation to treat various diseases associated with pathological tertiary lymphoid tissue formation [19–21]. Although agonistic or antagonistic activities of various anti-CD30 monoclonal antibodies are anticipated to be varied in an epitope-dependent fashion, the significance of the epitope location remains elusive because the previous structure–function analyses were complicated by the unusual structure of human CD30 containing large duplicate regions with two predicted ligand-binding sites. This structural complexity has prevented rational design and selection of both agonists and antagonists. An additional challenge in developing antagonistic antibodies is the bivalency of natural antagonistic antibody, which inevitably crosslinks trace amounts of target CD30 molecules, leading to signal transduction.

Herein, we focus on biparatopic antibodies (BpAbs), which are engineered bispecific antibodies using two different variable fragments (Fv) to two different epitopes of a single antigen molecule (Fig. 1B) [23, 24]. Recently, we successfully produced an antagonist against tumor necrosis factor receptor 2 (TNFR2), another member of the TNFRSF, using BpAb technology [25]. Similar to CD30, TNFR2 is activated by clustering upon binding to trimeric TNFα ligand. While binding by conventional bivalent antagonistic antibodies induced a moderate level of signaling in the absence of TNFα, the produced anti-TNFR2 BpAb antagonist bound TNFR2 in a 1:1 manner, resulting in no signal induction and maximizing antagonistic activity [25]. Building on this finding, we hypothesized that antagonists against TNFRSF members, including CD30, could be developed using a similar approach.

In this study, we selected a panel of nine previously made anti-CD30 mAbs whose topographical epitopes are distributed across the six CRDs on the extracellular domain of the human CD30 molecule [5, 26–29]. Since each pair of the nine antibodies recognize a unique set of topographical epitopes on CD30 molecules, we developed the biparatopic antibody series with all possible combinations of the nine antibody variable regions. We comprehensively evaluated the relationship of binding mode and CD30 downstream signaling potency. We identified both potent ligand-independent agonists and ligand-blocking antagonists. Among BpAbs exhibiting reduced agonistic activities, antagonistic BpAbs were identified, including those employing Fv from an agonist antibody in one arm, such as AC10. We discuss the insight obtained through the characterization of the binding modes in the contexts of the possible mechanism responsible for the signaling activities. In particular, we explore the molecular mechanisms underlying the conversion of agonists to antagonists. The overall data suggest that the targeted epitope can be selected to design BpAbs with desired functionality and different mode of actions for improved therapeutic activity.

## Results

### Nine anti-CD30 mAbs

We selected nine anti-human CD30 antibodies (T104, T6, T107, T427, T426, AC10, T105, T25, and T405) from a pool of previously known 42 antibodies. The nine mAbs bind distinct topographical epitopes spanning the native conformational structure of the extracellular domain of human CD30 [26–30]. Among them, AC10 is the original antibody used in Brentuximab vedotin [5]. Epitope information for the other antibodies not used in this study is summarized in Supplemental Text. The cDNA of Fv regions of these nine mAbs were obtained (Supplemental Table S1) and expressed as human IgG1κ chimera antibodies in conventional human IgG1 format (cAb). Competitive binding of the cAbs was analyzed in an enzyme-linked immunosorbent assay (ELISA) using CD30 fused to rabbit Fc (CD30-rFc) and in surface plasmon resonance (SPR) analysis using maltose-binding protein-fused CD30 (CD30-MBP; Supplemental Fig. S1). The ELISA-based assay (Table S2) and SPR analysis (Table S3 and Fig. S2) produced similar results showing the topographical relationships of the nine epitopes recognized by the cAbs, although slight differences were noted among duplicate-binding antibodies (T6, T107, and T427) [29], probably due to the use of dimeric antigen in the case of ELISA. In both cases, T426–T105, AC10–T427, and T427–T107 pairs showed complete competition. These findings were consistent with previously reported results [28–30].

### Ortholog screening and mutant analysis

Human CD30 is an unusual member of the TNFRSF with a duplicated segment within its extracellular domain. The extracellular region of human CD30 spans approximately 360 amino acids and contains six CRDs, commonly bound by specific ligands of the TNFRSF (Fig. 1C). Regions between CRD3 and CRD5, including the entire CRD4, are predicted to be disordered according to AlphaFold2 (Fig. 1D) [31, 32]. High homology regions exist within CRD1-3 and CRD4-6, likely due to gene duplication (Fig. 1E, F). These two regions exhibit functional similarity, with a common binding ability to CD153, the specific ligand for CD30 (Fig. 1G).

We initially aimed at precisely identifying amino acids residues associated with the epitope structures of the anti-CD30 antibodies. In an earlier report for another TNFRSF member, TNFR2, precise determination of epitope sites (down to approximately 2–10 amino acids) was conducted using chimeric mutants substituting human TNFR2 with mouse TNFR2 sequences [25]. However, in the case of CD30 in this study, similar epitope determination strategy based on human/mouse substitution posed challenges due to the duplicated region. Mouse CD30 (Uniprot ID: Q60846) possesses three CRDs only without the duplication compared to six CRDs in human CD30 with the duplicate region [33]. Therefore, human/mouse substitutions are not logically designed.

To address this issue, we screened ortholog genes of CD30 for similarity to human CD30. In a BLAST search of the protein sequence of the extracellular region [34], ortholog sequences showing 45–65% identities were selected. To ensure diversity among the orthologs, five sequences, spaced apart in a phylogenetic tree, were chosen (Fig. S3). These sequences correspond to predicted *tnfrsf8* gene products from *Tupaia chinensis* (*Tc*; Refseq ELW68632.1), *Chinchilla lanigera* (*Cl*; Refseq XP_013359778.1), *Lynx pardinus* (*Lp*; Refseq VFV35557.1), *Vicugna pacos* (*Vp*; Refseq XP_006197082.2), and *Myotis myotis* (*Mm*; Refseq KAF6380682.1) (amino acid sequences in Table S4). Structures of the CRDs of the ortholog *tnfrsf8* gene products were predicted using AlphaFold2 [31]. This allowed us focus on structurally similar protein domains suitable for substitution. CRD1, CRD2, CRD3, and CRD5 of human CD30 exhibit the typical CRD folding structure observed in TNFRSF proteins. In addition, the N-terminal half of CRD6 is folded. The structural similarity directly relates to protein function and, therefore, should provide a more reliable approach than random mutagenesis or alanine substitution. CRD1-3 of *Tc* are likely disordered, and CRD1 of *Cl* and *Vp* may also exhibit a different fold from CRD1 of humans (Fig. 1H). Structures of CRD5 and the N-terminal half of CRD6 (CRD5-6n) were predicted to be similar among the orthologs (Fig. 1I).

### Epitope determination

**Figure 2:**
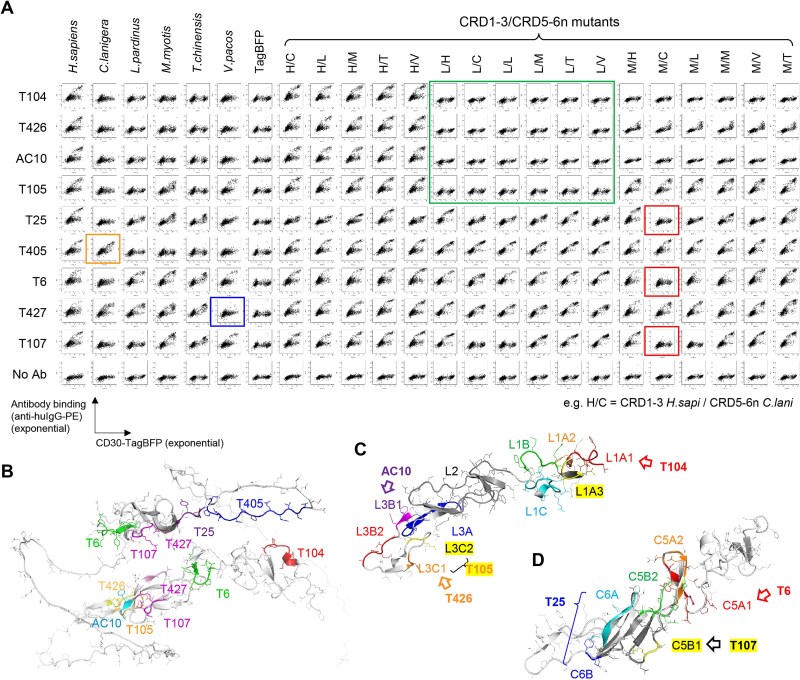
Epitope determination using CD30 mutants. (A) Affinity reduction of the antibodies with multi-domain substituted CD30 analyzed in flow cytometer. Right-ascending distribution indicates the presence of antibody binding, while horizontal distribution similar to “No Ab” indicates its absence. Green (L/H to L/V), red (M/C), blue (*V. pacos*), and orange (*C. lanigera*) boxes are key mutants or orthologs for epitope determination in the initial step. See also supplemental text. (B) Determined epitope sites of all antibodies. (C) CRD1-3 mutants used to determine epitopes of T104, AC10, T105, and T426. (D) CRD5-6n mutants used to determine epitopes of T6, T25, and T107.

All five gene products, transiently expressed in HEK293T cells, were bound by at least one of the nine antibodies (Fig. 2A), indicating expression of functional proteins on the cell membrane. Additionally, total or partial reduction in antibody binding levels were observed for each *tnfrsf8* gene product. Next, CRD1-3 was substituted with those from *Lp* and *Mm*, while CRD5-6n was substituted with those from all five orthologs. When antibody binding to 3 × 6 = 18 clones including human CD30 was analyzed (Table S4), several patterns of affinity reduction were observed (Fig. 2A). For epitope determination, further optimization of the mutants was conducted. Among the nine antibodies, three (T6, T107, and T427) were binders against the duplicate region. All mutants used in this study are listed in Table S4. The determined epitope regions are listed and mapped on the AlphaFold2-predicted structure of human CD30 (Table S5, Fig. 2B). Details are provided in the supplemental information (Supplemental Text, Figs S4–S9). In conclusion, the amino acid regions important for the epitope structures of T104, AC10, and T426 were L1A1, L3B1, and L3C1, respectively (Fig. 2C). T105 recognized both regions corresponding to L3C1 and L3C2 (Fig. 2C). The epitopes of T6 and T107 were MC5A1 and MC5B1, respectively (Fig. 2D). T25 recognized both regions corresponding to MC6A and MC6B (Fig. 2D). For T427, S88-D90/S263-D265 was found as the epitope core and S117-N120/S292-N295 in the periphery. For T405, the peptide D321-C335 was determined as the epitope.

### Production and binding activities of the BpAbs

Thirty-six BpAbs were produced from nine Fvs using a previously reported method to cover all biparatopic combinations of epitopes (Table 1) [35]. Although few fragments, such as T6^N^, showed low expression yields in the Expi293 expression system (Table S6), probably due to the poor folding ability of each Fv, *intein*-mediated protein trans-splicing (IMPTS) enabled homogeneous BpAb yield independent of the Fv used. The resulting BpAb by IMPTS is named after the identification numbers of the two Fvs, for example, BpT104-10 when T104^N^ and AC10^C^ are used (Table S7). The production method enabled high symmetry except for two amino acid substitutions in the hinge region of the “N” side Fab.

**Table 1:** Production of anti-CD30 BpAbs by the intein-fused materials used (such as T104^N^ and T426^C^).

“N” side	T104^C^	T426^C^	AC10^C^	T105^C^	T25^C^	T405^C^	T6^C^	T427^C^	T107^C^
T104^N^		BpT104-426			BpT104-25	BpT104-405	BpT104-6	BpT104-427	
T426^N^					BpT426-25	BpT426-405	BpT426-6	BpT426-427	
AC10^N^	BpT10-104	BpT10-426		BpT10-105	BpT10-25	BpT10-405	BpT10-6	BpT10-427	BpT10-107
T105^N^	BpT105-104	BpT105-426				BpT105-405	BpT105-6	BpT105-427	BpT105-107
T25^N^				BpT25-105		BpT25-405	BpT25-6	BpT25-427	
T405^N^							BpT405-6		
T6^N^									
T427^N^						BpT427-405	BpT427-6		
T107^N^	BpT107-104	BpT107-426			BpT107-25	BpT107-405	BpT107-6	BpT107-427	

Binding of the antibodies to the CD30-stably expressed NF-κB reporter Ramos-Blue cell line and the CD30-expressing lymphoma cell line KARPAS 299 [36] was analyzed. As expected, all cAbs and BpAbs showed binding to the CD30-Ramos-Blue cells in a dose-dependent manner (Fig. S10). All cAbs and BpAbs were also conformed with the reactivities with native CD30 molecule expressed on KARPAS 299 cell line (Fig. S11). The affinity of BpAbs as well as the original cAbs was analyzed in SPR at a rough level. In this experiment, antibodies were first captured on the sensor chip, and CD30-MBP was flowed next, minimizing the effect of avidity due to crosslinking patterns. As a result, all cAbs and BpAbs bound CD30-MBP with significant affinities, though the affinity of the T426 cAb was too weak to determine the parameters accurately (Fig. S12, Table S7). BpAbs, including T426-based ones, conferred strong affinity to CD30-MBP as typically observed for BpAbs [23]. The result indicated that both of Fvs comprising BpAbs play roles in the binding.

### Biological activities of the BpAbs

**Figure 3:**
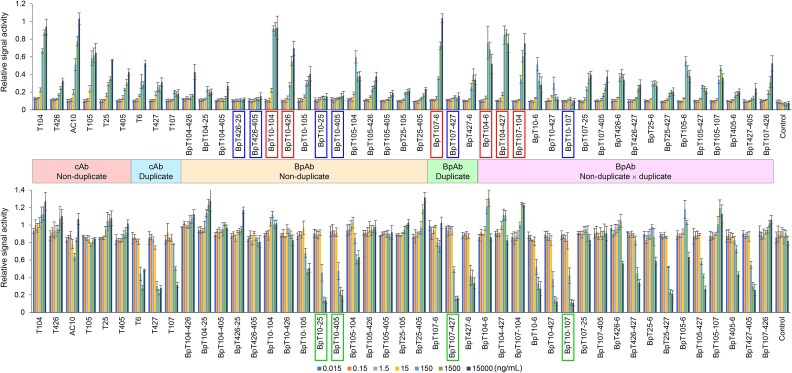
Agonistic and antagonistic activities of the cAbs and BpAbs analyzed using CD30-transfected NF-κB reporter cells. Agonistic (top) and antagonistic (bottom) activities were measured in the absence or presence of CD153-rFc. Antibodies are categorized by their recognizing non-duplicate and duplicate epitopes. Boxed labels indicate strong agonists (red), non-agonists (blue) and effective antagonists (green) among BpAbs. Unrelated anti-TNFR2 antibody TR109 [25] was used as the control.

The biological activities of the cAbs and BpAbs were analyzed using a Ramos-blue based reporter cell line that measures activation of NF-κB as the expression of secreted alkaline phosphatase [35]. Agonistic activity was analyzed by incubating cells with the antibodies in dilution series in the absence of the ligand, CD153 fused to rabbit Fc (CD153-rFc), whereas antagonistic activity was analyzed in the presence of CD153-rFc. The results are presented in Fig. 3. All nine cAbs demonstrated moderate to high levels of agonistic activities. Among them, AC10 showed the highest maximum agonistic activity reached, and another original cAb, T104, recognizing non-ligand binding site CRD1 showed a similar level of agonistic activity. In contrast, only a few cAbs showed antagonistic activity. The most potent antagonist was T427, but a significant level of residual signaling activity was observed, similar to findings for a different antibody specific to TNFR2 in a previous study [25].

BpAbs showed various profiles of activities. Six BpAbs (BpT10-104, BpT10-426, BpT107-6, BpT104-6, BpT104-427, and BpT107-104; highlighted in red, Fig. 3, top) showed maximum signaling activity comparable to AC10. As an agonist, BpT10-104 was superior to AC10 as it required a lower concentration to achieve this activity. Interestingly, BpT107-6 was a strong agonist even though the original cAbs (T107 and T6) were not. However, overall, BpAb offers only a limited level improvement in CD30 activation compared to cAbs.

**Figure 4:**
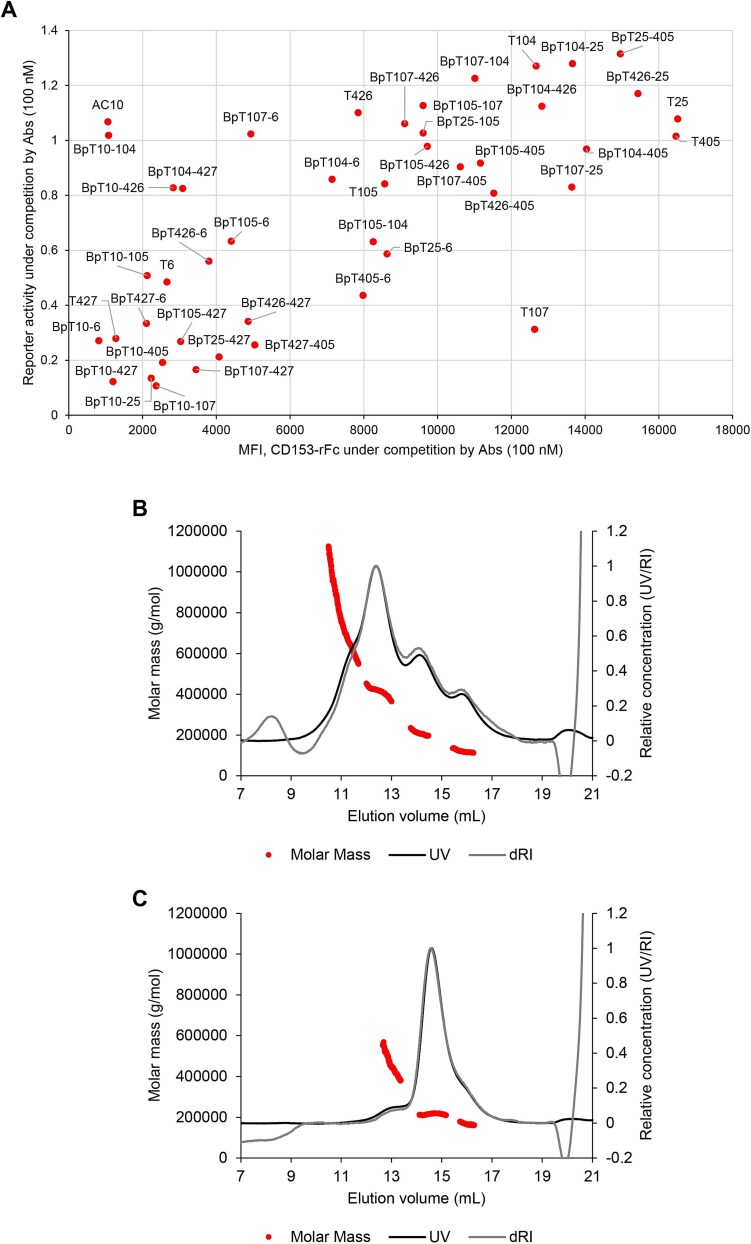
Binding characteristics of biparatopic antibodies. (A) Relationship between the biological antagonistic activity of the cAbs and BpAbs (*y*-axis) with CD153-binding competition to the CD30-expressing cells (*x*-axis). (B, C) Charts of size-exclusion chromatography–multiangle light scattering for BpT10-104 (B) and BpT10-405 (C) in complex with CD30-MBP. See Supplemental Fig. S14 for each component.

In contrast, improved antagonist candidates were generated using BpAb format. Six BpAbs (BpT426-25, BpT426-405, BpT10-25, BpT10-405, BpT107-427, and BpT10-107; highlighted in blue, Fig. 3, top) showed no agonistic activity. This elimination of agonistic activity of BpAb format led to the generation of BpAbs that showed strong antagonistic activities. Among cAbs, T427, T107 and T6 were potential antagonists. In the case of T6, activation of CD30 may offset its antagonistic activity when used at high concentration. BpAbs, including BpT10-25, BpT10-405, BpT10-427, BpT107-427, and BpT10-107 (highlighted in green, Fig. 3, bottom), showed stronger antagonistic functions, and apparent signaling activity was not observed. Interestingly, most BpAbs using AC10 Fvs were antagonists. Therefore, the most potent agonistic cAb AC10 was flipped into antagonist when used in BpAbs. To elucidate the mechanism of antagonistic activities, binding inhibition of CD153 ligand to the reporter cell line was analyzed in a flow cytometer (Fig. S13). The results showed that most antagonists acted as binding inhibitors of CD153 (Fig. 4A). AC10 also was a binding inhibitor of CD153, and thus, functionally hindered potential (ligand-blocking antagonism) arose as a function by building it into BpAbs. The reduction of agonistic activity through BpAb generation was key to the antagonistic function.

### Size of immunocomplexes

In a previous study, we developed anti-TNFR2 BpAbs that exhibited agonistic activities dependent on the size of immunocomplexes formed in solution with the soluble form of recombinant antigen proteins [25]. Using a similar method, we analyzed the size of immunocomplexes for agonistic and non-agonistic BpAbs using size-exclusion chromatography multiangle light scattering (SEC-MALS) (Fig. 4B, C, Fig. S14). To simplify the analysis, two BpAbs, BpT10-104 and BpT10-405, were selected that possess two Fvs binding to non-duplicate epitopes. The results showed that the agonist, BpT10-104, formed large immunocomplexes with the majority of 2:2 complexes (Fig. 4B, 12.3 ml peak, approximately 430 kDa), while the non-agonist, BpT10-405, only formed 1:1 immunocomplexes (Fig. 4C, 14.6 ml peak, approximately 220 kDa). Therefore, when non-duplicate epitopes were selected, the reduced agonistic activity correlated with a smaller size of the immunocomplexes. This result is consistent with our findings for the anti-TNFR2 BpAbs previously reported [25].

## Discussion

The TNFRSF encompasses a broad and varied array of cell surface receptors that play pivotal roles in governing immune responses, inflammation, and apoptosis. Recent research has emphasized the significance of receptor clustering in both activating and modulating TNFRSF receptor signaling [12–14, 37]. Malfunction in TNFRSF signaling is linked to various pathological conditions. Thus, controlling signaling has gained attention for therapeutic development [12, 13, 19, 20, 38]. Here, antibodies are considered promising [37]; however, rational design of either agonists or antagonists has remained challenging. Although recent studies suggest strategies to maximize biological activities through structural optimization of conventional antibodies [39–42], it is still difficult to design them based specifically on mechanistic insights. In this context, BpAbs present an attractive option for the control of signaling independent of the specific ligand [24, 25, 43].

For the rational development of BpAbs, understanding the mechanism of signal activation, specifically the relationship between structure and activity, is essential [23, 24]. The ability of cAbs and BpAbs to form TNFRSF clusters both in the presence or absence of the specific ligand affects signaling activity. We and others previously reported that the size of immunocomplexes is well associated with the ligand-independent signaling activity of agonistic BpAbs against TNFRSF members [25, 43]. In addition, ligand-independent signal activation, disadvantageous for developing antagonists, can be reduced by BpAb design [25].

**Figure 5:**
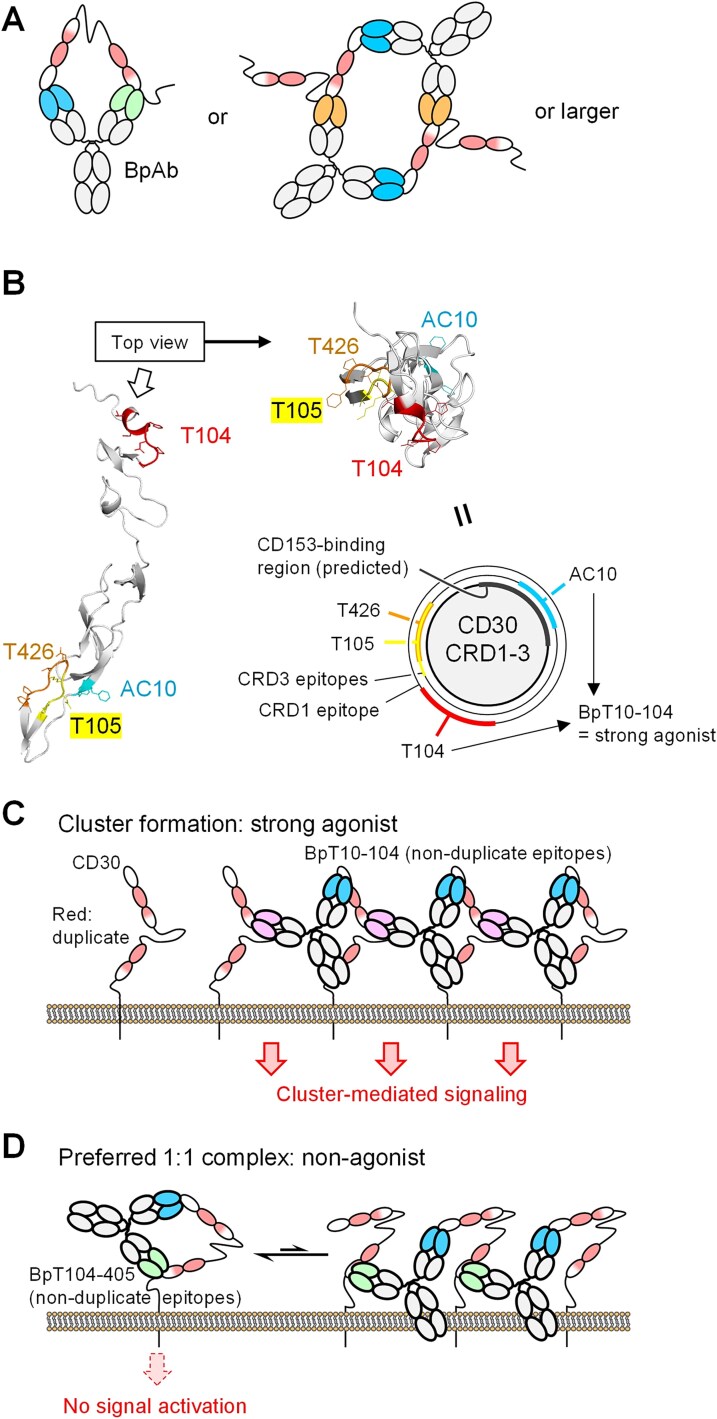
Immunocomplex formation by bivalent biparatopic antibodies (BpAbs) composed of two variable regions both binding non-duplicate epitopes. (A) Binding stoichiometry in a solution state model. Non-duplicate binding BpAbs form n:n (*n* = 1,2,3…) immunocomplex with the antigen. (B) Relationship between the positions of four CRD1-3 non-duplicate epitopes and agonistic activities of BpAbs. (C) BpT10-104, binding two regions in CRD1-3, favoring cluster formation. (D) Non-duplicate BpAbs divided by a flexible region, which prefer 1:1 immunocomplex formation and exhibit reduced agonistic activity.

To understand design strategy of anti-CD30 BpAbs as ligand-independent agonists and ligand-blocking antagonists, we first focus on BpAbs that bind to non-duplicate six epitopes to minimize the variations in the structure of immunocomplexes formed. Theoretically, they form n:n (*n* = 1,2,3…) complexes with the antigen when the epitope–paratope pairs are fully bound (Fig. 5A).

Among the six antibodies binding non-duplicate epitopes, four (T104, AC10, T426, and T105) bind to CRD1-3. For the six BpAbs produced from these four antibodies, cluster-forming ability reflecting the spatial location of the epitopes was expected. When viewed from the top of CRDs, the epitopes of AC10, T105, T426, and T104 are arranged in this order (Fig. 5B). We anticipated that the expected ligand-independent agonistic activity would be stronger for epitope pairs mapped father apart, as observed previously in the case of anti-TNFR2 BpAbs [25]. Indeed, the BpAb with the farthest pair of epitopes (AC10 and T104) exhibited the strongest signaling activity through cluster formation (Fig. 5C), as deduced from the observation in SEC-MALS (Fig. 4B). A similar activation mechanism of TNFRSF members by BpAbs was suggested [25, 43]. It should be noted that Fv combinations too close to each other, such as T426 and T105, resulted in competition for binding (Tables S2 and S3), therefore, the binding mode in these cases was similar to that of cAbs. The other BpAbs demonstrated moderate agonistic activity, although it is challenging to discern slight differences among them.

On the other hand, the other two non-duplicate antibodies (T25 and T405) bind to the epitopes in CRD6. The nine BpAbs that incorporate one of the Fvs from T25 or T405 were not strongly agonistic. In particular, BpT426-25, BpT426-405, BpT10-25, and BpT10-405 lacked agonistic activity in the absence of CD153-rFc. Based on the analysis of BpT10-405 in SEC-MALS, these BpAbs preferred the formation of 1:1 immunocomplexes (Fig. 4C). Possibly because of the flexible region between the two epitopes, simultaneous binding of one Fv recognizing CRD1-3 region and the other Fv recognizing CRD6 in the same molecule would be promoted (Fig. 5D). Since AC10 blocks CD153 binding (Fig. 4A), BpT10-25 and BpT10-405 exhibited strong antagonistic activity, functioning as pure antagonists similarly to a BpAb against TNFR2, as we previously reported [25]. Antagonists based on this mechanism were not found among cAbs, highlighting the advantage of developing BpAbs for binding without cluster formation. To summarize, biological activities and immunocomplex forming patterns of non-duplicate binding BpAbs are well interpreted and predictable.

**Figure 6:**
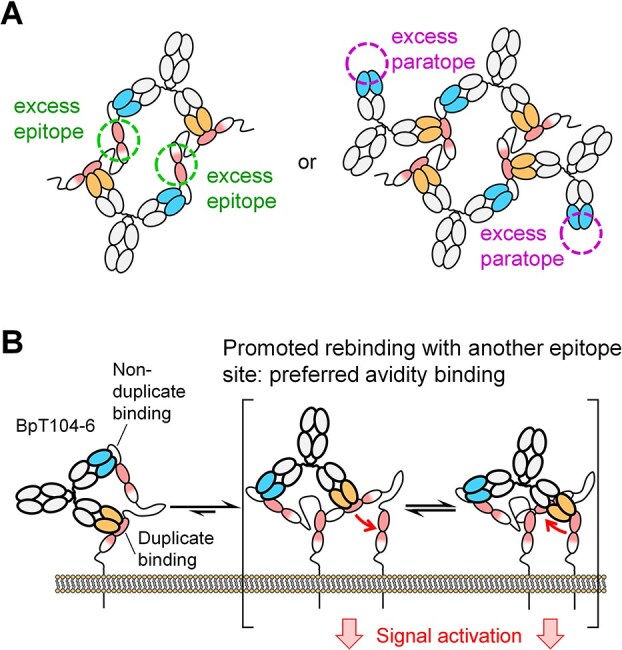
Immunocomplex formation by bivalent biparatopic antibodies (BpAbs) composed of two variable regions each binding duplicate and non-duplicate epitopes. (A) Binding stoichiometry in a solution state model. Because the number of epitopes is different, either epitopes or paratopes are always excessive and remain unbound. (B) Putative mechanism of duplicate-binding BpAbs showing moderate agonistic activity. Due to proximity, rebinding to the unbound epitope is promoted after dissociation of duplicate-binding Fv, which may function in a crosslinking-like binding among multiple CD30 molecules rather than binding to a single CD30 molecule.

In contrast, the other 21 duplicate-binding BpAbs exhibited complicated activation patterns in the absence of CD153-rFc. Particularly, BpAbs that utilize one Fv from the duplicate binder and another Fv from the non-duplicate binder may exhibit complex binding patterns due to the imbalance in the number of two epitope–paratope pairs. As depicted in Fig. 6A, either epitopes in the duplicate region of CD30 or paratopes of BpAbs binding the non-duplicate region are always excessive. The possible binding patterns are numerous, making it challenging to generate a comprehensive model. There was no simple correlation with the relative position of epitopes in the activity. Only two non-agonists (BpT10-107 and BpT107-427) were found among these BpAbs. We originally expected that BpAbs with at least one Fv binding the duplicate region would favor a 1:1-binding mode, resulting in most of these BpAbs functioning as non-agonists similar to the mechanism in Fig. 5C. Experimental observation, however, contradicted this prediction, suggesting that most of these BpAbs favor binding multiple CD30 molecules. The mechanism may be partly explained by promoted rebinding after dissociation between two duplicate epitopes in the same molecule, resulting in energetically favored avidity binding advantageous for a crosslinking-like immunocomplex formation (Fig. 6B). Agonists among these BpAbs showed bell-shaped response (e.g. BpT104-6 and BpT104-427 in Fig. 3, upper charts), which also supports the roles of excess epitopes or paratopes (Fig. 6). These observations highlight the challenges of designing functional BpAbs targeting duplicate regions of CD30. In addition, duplicate-binding cAbs may be governed in a similar mechanism, which may explain the absence both of strong agonists and non-agonists.

Findings of the present study are similar to our previous work targeting TNFR2 [25]. We identified potential ligand-blocking antagonists among 1:1-binding BpAbs, where two different Fvs bind different epitopes, which do not induce signal activation in the absence of a specific ligand. Such effective antagonists were not observed among cAbs. A significant revelation was the discovery of antagonistic activity in AC10-based BpAbs. AC10, the strongest agonistic cAb among those analyzed, exhibited maximum signal activation surpassing even the strongest agonistic BpAb. However, by transitioning into a BpAb format, its potential to block CD153 was fully utilized by minimizing crosslinking. It is noteworthy that modifying activity based on the flexible nature of CD30 is possible by using a BpAb format.

In summary, we developed 36 BpAbs from nine Fvs binding different epitopes of CD30, including three Fvs binding the duplicate region. We identified several BpAbs as non-agonists and successfully, developed strongly antagonistic BpAbs among CD153-competitive BpAbs. These BpAbs included those utilizing the Fv sequence from a potent agonist, AC10. Both for ligand-independent agonists and antagonists, duplicate-binding epitopes should be avoided. The mechanism underlying reduced agonistic activity is similar to that observed in TNFR2, another member of TNFRSF, which forms BpAb:antigen = 1:1 immunocomplexes. These findings pave the way for the development of CD30 antagonists and also contribute to the design of antagonistic BpAbs against association-activated receptors.

## Materials and methods

### Cell culture

HEK293T cells were sourced from the American Type Culture Collection and maintained in DMEM supplemented with 10% fetal bovine serum (FBS). KARPAS 299 cells were obtained from the German Collection of Microorganisms and Cell Cultures (DSMZ, Braunschweig, Germany). Ramos-Blue cells were purchased from InvivoGen (San Diego, CA, USA) and cultured in IMDM supplemented with 10% FBS. The cells were further transfected in our laboratory with pcDNA6-derived plasmids encoding full length of CD30 followed by selection with blasticidin-containing medium.

### Production of recombinant antibodies

Recombinant antibodies were produced according to established protocols [25, 35]. Conventional antibodies (cAbs) were generated as human IgG1κ chimera antibodies in this study. The expression and purification of BpAbs followed previously described a series of methods, which are based on IMPTS. Briefly, Fab “**N**” was fused to Cfa Int^N^-MBP, and Fab “**C**” was fused to an Fc with a “knob” mutation using the knobs-into-holes method. Fab “**C**” was coexpressed with an MBP-Cfa Int^C^ fusion of Fc with a “hole” mutation to produce a monovalent antibody. Following IMPTS, the BpAbs were purified using a Superdex 200 Increase 10/300 GL column (Cytiva).

### Preparation of recombinant CD30 and CD153 proteins

CD30 whole extracellular domains (NP_001234.3, amino acids 1-383), or CD30 fragments (CRD4, CRD1-3, or CRD4-6) C-terminally fused to the hinge and Fc portion of human or rabbit IgG1 were cloned into pcDNA3 plasmid (InVitrogen). The fusion proteins were harvested from the culture supernatant of HEK293T cells transfected with the plasmid [44]. CD30 extracellular domains (1-336) C-terminally fused to MBP (CD30-MBP; Supplemental Fig. S1) were cloned into pcDNA3.1. The protein was expressed in Expi293 Expression System. The culture supernatant was dialyzed overnight against Buffer A (20 mM Tris, 300 mM NaCl, pH 8.0) containing 5 mM imidazole. Expressed proteins were captured on Ni-NTA Superflow (Qiagen) and washed with Buffer A containing 5, 10, and 20 mM imidazole, and the protein was eluted using Buffer A containing 200 mM imidazole. The eluate was dialyzed against phosphate-buffered saline (PBS), and the final purification was conducted using a Superdex200 16/600 column.

CD153 extracellular region (NP_001235.1, Gln63-Asp234) was N-terminally fused to Fc portion of rabbit IgG and subcloned into pSecTag2 Hygro A plasmid (InVitrogen). The Fc-fused proteins (CD153-rFc) were expressed in HEK293T cells and purified by protein A affinity chromatography as described previously [26].

### Mutual competitive binding assay of cAbs

All pairs of the 9 cAbs (9 × 9) were tested with the sequential binding activity for the binding to CD30-rFc protein in an ELISA as described previously with slight modifications [25, 28]. Microplates were coated with goat antihuman IgG Fc (#109-005-098, Jackson Immunoresearch) and each indicator cAb (0.75 μg/mL) was captured. Competitor cAb (2.8 μg/mL) and CD30-rFc (50 ng/mL) were incubated overnight at 4 °C in a separate tube. After washing the plates, the competitor–CD30-rFc solution was added to each well of the coated plates. After washed twice, bound CD30-rFc was probed by alkaline phosphatase-conjugated goat antirabbit IgG (#111-055-046, Jackson Immunoresearch).

For SPR-based analyses, F(ab′)_2_ protein was produced by digestion using 10% (w/w) IdeS protease on each cAb. His-tagged IdeS protease and digested Fc were removed by NEBExpress Ni Spin Columns (New England Biolabs) and Protein A HP SpinTrap (Cytiva), respectively. cAbs and CD30-MBP were successively captured as described above, and the interaction of F(ab′)_2_ (20 nM) was analyzed by the contact and dissociation time of 90 and 120 s, respectively, flowed at 30 μl/min.

### Binding analysis of CD153 to CD30

Microplates were coated with goat anti-human IgG Fc antibody (#109-005-098, Jackson Immunoresearch) for 2 h at room temperature. After blocking and washing, to appropriate wells we added 200 ng/100 μl/well of each CD30-deletion mutant-human Fc in blocking buffer. After washing, CD153-rFc in concentration series was added. HRP-goat-anti-rabbit IgG Fc antibody (#111-035-046, Jackson Immunoresearch) was used to detect the bound CD153-rFc.

### CD30 orthologs

Ortholog species containing the *tnfrsf8* gene with moderate structural homology to human CD30 (Refseq NP_001234.3) were screened. Homologous sequences within the extracellular region of human CD30 (F19-G385 as defined in UniProt) were identified using protein BLAST [34]. Seven sequences with approximately 60% homology to human CD30 (Refseq XP_022454406.1, XP_036855465.1, XP_014705247.1, KAF6380682.1, VFV35557.1, XP_006197082.2, XP_013359778.1) and five sequences with approximately 50% homology to human CD30 (Refseq XP_036084707.1, XP_027474377.1, XP_007533056.1, XP_034527286.1, ELW68632.1) were selected. Among the 12 sequences, five with distinct distances from each other in the phylogenetic tree generated using Clustal 2.1 were further chosen for mapping antibody binding [45]. DNA encoding the full-length TNFRSF8 proteins of five selected orthologs was synthesized by Genewiz LLC. The predicted human CD30 structure was obtained from AlphaFold DB (AF-P28908-F1) [32]. Structures of CD30 orthologs from other species were predicted using AlphaFold2 in ColabFold v1.1 with the MSA mode of MMSeq2 [31, 46]. One of the five generated models was visualized using Pymol (Schrödinger, LLC).

### Ortholog and epitope mapping

DNA encoding human and the five ortholog sequences were fused with TagBFP on the C-terminus and cloned into pcDNA3.1. The sequences of the encoded proteins used in the ortholog and epitope mapping are described in Table S4. The expression vector was transfected into HEK293T cells using PEI “MAX” (Polysciences Inc.). Cells were cultured for 40 h posttransfection and detached from the culture vessels using trypsin/EDTA for 5 min. Cells were covalently labeled with combinations of succinimidyl ester compounds of Pacific Orange, DyLight 633, or DyLight 800 (Cat. No. P30253, P46414, and P46421; Thermo Fisher Scientific), as described previously [25]. Twelve differently labeled cells at maximum per experiment were combined.

### Flow cytometry

For antibody binding, mutant-transfected HEK293T cells, KARPAS 299 cells or CD30-RB cells (1 × 10^5^ cells/well) were incubated with each antibody for 30 min on ice. After being washed twice, the cells were further incubated with the secondary antibody antihuman IgG, Fcγ-PE (1/200 dilution, #109-116-170; Jackson Immunoresearch) for 30 min on ice. For CD153-binding competition, CD30-RB cells were incubated with a mixture of the antibodies (1.5 μM) and CD153-rFc (200 ng/ml) for 30 min on ice, washed twice, and further incubated with the secondary antibody anti-rabbit IgG-PE (1/200, #111-116-144; Jackson Immunoresearch). The cells were then analyzed using a BD LSRFortessa Cell Analyzer. Data were analyzed in FlowJo v10.10.0.

### Reporter assay

CD30-expressing Ramos-Blue (CD30-RB) cells were seeded at 5 × 10^4^ in 100 μl of medium-containing antibodies at the indicated concentrations (100 fM to 100 nM in tenfold dilution series) in the absence or presence of 200 ng/ml CD153-rFc. The cells were incubated for 18 h, and secreted alkaline phosphatase was analyzed using *p*-nitrophenyl phosphate. Colorimetric changes were determined by measuring absorbance at 405 nm using an EnSpire microplate reader (PerkinElmer). Signals were normalized to the average absorbance of the eight wells incubated without antibody or CD153-rFc (negative) and the eight wells incubated only with 200 ng/mL CD153-rFc (positive). For the chart showing the agonistic and antagonistic activities, the negative and positive values were set at 0.1 and 0.9, respectively.

### Binding affinity analysis to recombinant CD30 protein in SPR

The binding kinetics of an antibody–antigen interaction was measured using a Biacore T200 instrument (Cytiva). In brief, antihuman Fc was immobilized on a CM5 chip using the Human Antibody Capture Kit (Cytiva). To immobilize anti-human Fc (approximately 7000 RU), cAbs and BpAbs (1 μg/mL) were capture by flowing at 10 μL/min for 300 s (T25 and BpT104-405) or 60 s (others). The interaction with CD30-MBP was analyzed by the contact and dissociation time of 90 and 300 s, respectively, flowed at 30 μ/min. The concentration of CD30-MBP was 0, 20, and 200 nM for analyzing T426 and 0, 2, and 20 nM for analyzing others.

### SEC-MALS

SEC-MALS measurements were conducted, as previously described [25]. Briefly, each antibody and CD30-MBP were mixed equimolarly (2 μM) in PBS, and a 50 μLlsolution was loaded onto a Superose 6 Increase 10/300 GL column (Cytiva) column. Light scattering was detected in DAWN 6 (Wyatt, Santa Barbara, CA, USA). Data were analyzed using ASTRA 6 software (Wyatt). The protein concentration was calculated from the refractive index using dn/dc = 0.175. Molar mass values were determined by the Debye fitting of angle-dependent light scattering.

## Supplementary Material

Akiba_SI_revision_tbaf002

Supplementary_Table_S4_tbaf002

## Data Availability

All data supporting the findings of this study are available within the article and Supplemental Information. Raw data files are available by reasonable request to the corresponding authors.
