# Supplementary material for: Generation of antagonistic biparatopic anti-CD30 antibody from an agonistic antibody by precise epitope determination and utilization of structural characteristics of CD30 molecule
Source: Antib Ther. 2025 Jan 14;8(1):56–67. doi: 10.1093/abt/tbaf002 (PMC11826918; doi:10.1093/abt/tbaf002)
Supplement: Akiba_SI_revision_tbaf002 [file akiba_si_revision_tbaf002.pdf]

Supplemental Information for
Generation of antagonistic biparatopic anti-CD30 antibody from an agonistic antibody by precise epitope determination and utilization of structural characteristics of CD30 molecule

Hiroki Akiba[1,2]*, Tomoko Ise[3], Reiko Satoh[2], Yasuhiro Abe[4], Kouhei Tsumoto[5,6], Hiroaki Ohno[1,2], Haruhiko Kamada[1,2], Satoshi Nagata[3]*

1 Graduate School of Pharmaceutical Sciences, Kyoto University

2 Laboratory of Advanced Biopharmaceuticals, Center for Drug Design Research, National Institutes of Biomedical Innovation, Health and Nutrition

3 Laboratory of Antibody Design, Center for Drug Design Research, National Institutes of Biomedical Innovation, Health and Nutrition

4 Division of Drugs, National Institute of Health Sciences

5 School of Engineering, The University of Tokyo

6 The Institute of Medical Science, The University of Tokyo

*Corresponding Authors.

Email: hakiba@pharm.kyoto-u.ac.jp (H.A.); Satoshi-Nagata@nibiohn.go.jp (S.N.)

Table of Contents	Page #
Supplemental Text	S2-5
Table S1–S7*	S6-11
Fig. S1–S14	S12-27

* Table S4 is not included in this file and provided as a separate file.

Information of epitopes of anti-CD30 antibody clones unused in this study

In the present study, a panel of nine MAbs to nine distinct topographical epitopes on the conformational structure of the extracellular domain of CD30 was selected from a set of 42 mAbs previously characterized. The selection process was informed by existing data on the 42 known mAbs and involved eliminating redundant mAbs targeting the same epitopes. The epitope locations of the mAbs were defined relative to other anti-CD30 mAbs by competition assays using CD30-expressing cells or CD30-Fc fusion proteins, as documented in the following previous reports.

Twelve reference mAbs were assigned to human CD30 in previous Human Leucocyte Differentiation Antigens (HLDA) workshops (1). Out of these 12, the AC10 clone was included in the final panel of nine mAbs in this study. The clones Ki-I (2), Ber-H2 (3) and HRS-4 (4) were evaluated by us for the relative epitope location alongside the nine selected mAbs using 28 different mAbs (5-7). Additionally, HRS-1 (8) and HRS-3 were reported to bind to the same epitope group as Ber-H2 and HRS-4 (4), while Ber-H8 was found to recognize the same epitope for Ber-H2 (9). Ber-H6 and Ber-H10 bind to the 1-93 amino acid region as Ki-I (10), and BerH4 and Ber-H8 react with 112-412 amino acid region (10). The HeFi-I clone (11) is one of the initially isolated anti-CD30 mAbs and was used in a past phase I clinical trial (ClinicalTrials.gov Identifier: NCT00048880). HeFi-I has often been used as a reference antibody in studies to evaluate the topographical locations of other CD30 epitopes. HeFi-I binding to CD30 was reported to be inhibited by AC10 (12). Clones M44 and M67 (13), and clones Ki-2, Ki-3, Ki-4, Ki-5, Ki-6 and Ki-7 (12) were produced separately. A comprehensive epitope mapping study (12) indicated that (i) Ki-2, Ki-4, Ki-6 and Ki-7 recognize the same topographical epitope as Ber-H2, HRS-1 and HRS-4; (ii) M67 and Ki-5 bind to an epitope group defined by Ki-I; (iii) M44 and Ki-3 bind to the same epitope as AC10 and HeFi-I. A portion of these epitope mapping results was corroborated by another study (9). The epitopes defined for M67 and Ki-4 were included in the nine topographical epitope groups based on our experiments (5-7). Another human anti-CD30 mAb, 5F11, was generated in the HuMAb mouse (14), and was used in clinical trials. 5F11 binds to the topographical epitope defined by Ber-H2, Ki-4, Ki-2 and HRS-3, and the location is distinct from the epitopes of Ki-1or of AC10 and Ki-3 (14). We have produced 23 anti-CD30 mAbs in 6 experiments (6,7).

Detailed description of epitope determination for each antibody.

(1) CRD1-3 binders, including T104, AC10, T426, and T105. These four antibodies showed a common binding pattern independent of CRD5-6n (Fig. 3A). They exclusively

recognized CRD1-3. Since all antibodies failed to bind to *Lp*CRD1-3 (green box in Fig. 3A), the critical amino acid residues for constituting the epitope structures were narrowed down by partial substitution to *Lp* sequence. Initially, each CRD was substituted. T104 did not bind to the CRD1 mutant of *Lp* (denoted as L1), while the other three antibodies did not bind to the CRD3 mutant of *Lp* (L3) (Fig. S4). For T104, CRD1 was further split into three based on disulfide connectivity (L1A, L1B, and L1C), and L1A was not bound (Fig. S5). By further narrowing down the mutated region (L1A1, L1A2, and L1A3), a reduction in binding to L1A1 was observed, and this region was assigned as the critical amino acids for the epitope structure (Fig. S5). In a similar strategy, CRD3 was split into three (L3A, L3B, and L3C), and then into two pairs (L3B1 and L3B2, L3C1, and L3C2). Binding was reduced for L3B1 by AC10, and L3C1 by T426 (Fig. S6). Binding of T105 was not observed for L3C, but the split mutants were bound; thus, both regions corresponding to L3C1 and L3C2 contributed partly to the binding of T105 (Fig. 3C, Fig. S6).

(2) T6, T107 and T25. These antibodies exhibited reduced binding by *Mm*CRD1-3 and *Cl*CRD5-6n doubly substituted clone (red boxes in Fig. 3A). Based on this, a partial *Cl* mutant was introduced to CRD5 or CRD6 of *Mm*CRD1-3, and a similar method was conducted as in *(1)* to narrow down the epitopes. As a result, T6 showed reduced binding to the partial mutant of CRD5 (MC5A1), and T107 similarly showed reduced binding to MC5B1. T25, on the other hand, exhibited only a partial reduction in binding to MC6A. When the adjacent region was also substituted with *Cl* sequence (denoted as MC6B), a reduction in the affinity was observed (Fig. 3D, Fig. S7). Therefore, this region was determined as the epitope.

(3) T427. The epitope of T427 was previously reported to be inside the duplicate region (15). Among the tested ortholog clones, only *Vp* showed reduced binding (blue box in Fig. 3A). When all CRDs in this region of human CD30 were substituted with the *Vp* sequence (V2356), no binding was observed (Fig. S8). By comparing the peptide sequences to search for regions with multiple amino acid differences between *Vp* and human CRD2-3 and CRD5-6, three pairs of regions were found (Fig. S8). These regions inside CRD2-3 of V2356 were back-mutated to the human sequence (named V2356-EAD, V2356-SRD, V2356-SAV). The SRD mutation fully restored binding, the SAV

mutation partially, while the EAD mutation did not restore binding (Fig. S8). Thus, S88-D90/S263-D265 was found as the epitope core and S117-N120/S292-N295 in the periphery.

(4) T405. The epitope of T405 was reported to be inside CRD6 (25). Among the ortholog sequences, only *Cl* showed binding (orange box in Fig. 3A), thus, the region that only *Cl* shows high homology in the C-terminal disordered region of CRD6 was the candidate. This coincided with D321-C335 (Fig. S9). When these regions were substituted with *Mm* sequence (M6e), or split in half (M6e1 and M6e2), all showed reduced binding (Fig. S9). Thus, the epitope of T405 was determined to be this whole region.

References

1. Schwarting, R., and Stein, H. (1989) Cluster report: CD30. in *Leukocyte Typing IV White Cell Differentiation Antigens* (Knapp, W., Dörken, B., Gilks, W. R., Rieber, E. P., Schmidt, R. E., Stein, H., and Borne, A. E. G. K. v. d. eds.), Oxford University Press, New York. pp 419-427
2. Schwab, U., Stein, H., Gerdes, J., Lemke, H., Kirchner, H., Schaadt, M., and Diehl, V. (1982) Production of a monoclonal antibody specific for Hodgkin and Sternberg-Reed cells of Hodgkin's disease and a subset of normal lymphoid cells. *Nature* 299, 65-67
3. Schwarting, R., Gerdes, J., Durkop, H., Falini, B., Pileri, S., and Stein, H. (1989) BER-H2: a new anti-Ki-1 (CD30) monoclonal antibody directed at a formol-resistant epitope. *Blood* 74, 1678-1689
4. Engert, A., Burrows, F., Jung, W., Tazzari, P. L., Stein, H., Pfreundschuh, M., Diehl, V., and Thorpe, P. (1990) Evaluation of ricin A chain-containing immunotoxins directed against the CD30 antigen as potential reagents for the treatment of Hodgkin's disease. *Cancer Res*. 50, 84-88
5. Soler-García, A., Maitra, R., Kumar, V., Ise, T., Nagata, S., Beers, R., Bera, T., and Pastan, I. (2005) The PATE gene is expressed in the accessory tissues of the human male genital tract and encodes a secreted sperm-associated protein. *Reproduction* 129, 515-524
6. Nagata, S., Numata, Y., Onda, M., Ise, T., Hahn, Y., Lee, B., and Pastan, I. (2004) Rapid grouping of monoclonal antibodies based on their topographical epitopes by a label-free competitive immunoassay. *J. Immunol. Methods* 292, 141-155
7. Nagata, S., Onda, M., Numata, Y., Santora, K., Beers, R., Kreitman, R., and Pastan, I. (2002) Novel anti-CD30 recombinant immunotoxins containing disulfide-stabilized Fv fragments. *Clin. Cancer Res.* 8, 2345-2355
8. Pfreundschuh, M., Mommertz, E., Meissner, M., Feller, A. C., Hassa, R., Krueger, G. R., and Diehl, V. (1988) Hodgkin and Reed-Sternberg cell associated monoclonal antibodies HRS-1 and HRS-2 react with activated cells of lymphoid and monocytoid origin. *Anticancer Res.* 8, 217-224
9. Franke, A. C., Jung, D., and Ellis, T. M. (2000) Characterization of the CD30L binding domain on the human CD30 molecule using anti-CD30 antibodies. *Hybridoma* 19, 43-48

10. Falini, B., Pileri, S., Pizzolo, G., Durkop, H., Flenghi, L., Stirpe, F., Martelli, M. F., and Stein, H. (1995) CD30 (Ki-1) molecule: a new cytokine receptor of the tumor necrosis factor receptor superfamily as a tool for diagnosis and immunotherapy. *Blood* 85, 1-14

11. Hecht, T. T., Longo, D. L., Cossman, J., Bolen, J. B., Hsu, S. M., Israel, M., and Fisher, R. I. (1985) Production and characterization of a monoclonal antibody that binds Reed-Sternberg cells. *J. Immunol.* 134, 4231-4236

12. Horn-Lohrens, O., Tiemann, M., Lange, H., Kobarg, J., Hafner, M., Hansen, H., Sterry, W., Parwaresch, R. M., and Lemke, H. (1995) Shedding of the soluble form of CD30 from the Hodgkin-analogous cell line L540 is strongly inhibited by a new CD30-specific antibody (Ki-4). *Int. J. Cancer* 60, 539-544

13. Gruss, H. J., Boiani, N., Williams, D. E., Armitage, R. J., Smith, C. A., and Goodwin, R. G. (1994) Pleiotropic effects of the CD30 ligand on CD30-expressing cells and lymphoma cell lines. *Blood* 83, 2045-2056

14. Boll, B., Hansen, H., Heuck, F., Reiners, K., Borchmann, P., Rothe, A., Engert, A., and Pogge von Strandmann, E. (2005) The fully human anti-CD30 antibody 5F11 activates NF-κB and sensitizes lymphoma cells to bortezomib-induced apoptosis. *Blood* 106, 1839-1842

15. Nagata, S., Ise, T., Onda, M., Nakamura, K., Ho, M., Raubitschek, A. and Pastan, I.H. (2005) Cell membrane-specific epitopes on CD30: Potentially superior targets for immunotherapy. Proc. Natl. Acad. Sci. U. S. A., 102, 7946-7951

Table S1. Amino-acid sequences of variable regions from nine antibodies used in this study

	VH	VL
**T104**	QIQLVQSGPELKKPGETVKISCKASGYTFTDYSMHWVKQAPGK<br>GLKWMGWINTETGEPTYADDFKGRFAFSLETSASTAYLQINNL<br>KNEDTATYFCTEGYVFDFWGQGTTLTVSS	DILLTQSPAILSVSPGERVSFSCRASRSIGTSIHWYQQRTNGS<br>PRLLINYASESISGIPSRFSGSGSGTDFTLSINSVESEDIADY<br>YCQQSNSWPLTFGAGTKLELKR
**T426**	EVQLQQSGAELVKPGASVKLSCTASGFNVKDTYVHWVKQRPEQ<br>GLEWIGRIDPANSDTKYDSKFQGKATTSADTSSNTAYLQLNSL<br>TSEDTAVYYCASGTDDYYWYFDVWGAGTTVTVSS	DIVLTQSPASLAVSLGQRATISCRASKSVSTPAHTYMHWYQQK<br>PGQPPKLLIYLASNLESGVPARFSGSGSGTDFTLNIHPVEVED<br>AATYYCQHSRVLPYTFGGGTKLEIKR
**AC10**	QIQLQQSGPEVVKPGASVKISCKASGYTFTDYYITWVKQKPGQ<br>GLEWIGWIYPGSGNTKYNEKFKGKATLTVDTSSSTAFMQLSSL<br>TSEDTAVYFCANYGNYWFAYWGQGTQVTVSA	DIVLTQSPASLAVSLGQRATISCKASQSVDFDGDSYMNWYQQK<br>PGQPPKVLIYAASNLESGIPARFSGSGSGTDFTLNIHPVEEED<br>AATYYCQQSNEDPWTFGGGTKLEIK
**T105**	QVTLKESGPGILQPSQTLSLTCSFSGFSLSTSGMGVSWIRQPS<br>GKDLEWLAHIYWDDDKRYNPSLKSRLTISKDTSSNQVFLKITS<br>VDTADTATYYCARRADGLYFYLDVWGAGTTVTVSS	DIVMTQSQKFMSTSVGDRVSVTCKASQNVNTNVAWYQQKPGQS<br>PEALIYSASYRYSGVPDRFTGSGSGTDFTLTISNVQSEDLAEY<br>FCQQYNSYPLTFGSGTKLEIK
**T25**	QVTLKESGPGILQPSQTLSLTCSFSGFSLNTSGVGVGWIRQPS<br>GKGLEWLAHIWWDDDERYNPVLKSRLTISKDTSSNQVFLKIAN<br>VDTADSATYYCVRSMVAWFPYWGRGTLVTVSA	DIVMSQSPSSLAVSVGEKFTVNCKSSQSLLYSSNQKNFLAWYQ<br>QKPGQSPKLLIYWASTRESGVPDRFIGSGSGTDFTLTISSVKA<br>EDLAVYYCQQHYRYPWTFGGGTKLEIK
**T405**	QVQLQQIGAELVRPGASVKLSCKASGYTFNNYWINWVKQRPGQ<br>GLEWIGNIYPSDSRSNYNQKFKDKATLTVDKPSSTAYMQLSSP<br>TSEDSAVYYCTLGSYWGQGTLVTVSA	DVVMTQTPLTLSVTIGQPASISCKSSQSLSDSDGKTYLNWLLQ<br>RPGQSPKRLIYLVSKLDSGVPDRFTGSGSGTDFTLKISRVEAE<br>DLGVYYCWQGAHFPRTFGGGTKLEIK
**T6**	QVQLKESGPGLVTPSQSLSITCTVSGFSLSKYSIHWVRQPPGR<br>GLEWLGMIWGVENTDYNSALKSRLSISKDNSKSQVFLKMNSLQ<br>SDDTAMYYCARKDLGLYGMNYWGQGISVTVSA	DIVMTQSQKFMSTSVGDRVSVTCKASQNVGTNVAWYQQKPGQS<br>PKALFYSASYRYSGVPDRFIGSGSGTVFTLTISNVQSEDLAEY<br>FCQQYNTYPLTFGSGTKLEIE
**T427**	QVQLQQPGTELVRPGASVKLSCKASGFSFTSYWMNWVKQRPGQ<br>GLEWIGMIHPSDSETRLNQKFKDRATLTVDKSSSTAYMQLSSP<br>TSEDSAVYYCASEMDYYFAMDYWGQGTSVTVSS	DIVLTQSPTSLAVSLGQRATISCRASESVDSYGNSFMHWFQQK<br>PGQPPKLLIYRASNLESGIPARFSGSGSWTDFTLTINPVEADD<br>VATYYCQQSNEDPRTFGGGTKLEIKR
**T107**	QVQLQQSGPELVRPGVSVKISCKGSGYSFPDYPLHWVKQSHAK<br>SLEWIGIILTYSGNTNYNQNFKGKATMTVDTSSNTAYLELARL<br>TSEDSAIYYCATFYYGYDGAFDYWGQGTTLTVSS	DIVMTQSPSSLTVTAGEKVTMSCKSSQSLLNSGNQKNYLTWYQ<br>QKPGQPPKLLIHWASTRESGVPDRFTGSGSGTDFILTITSVQA<br>EDLAVYYCQNDYTYPLTFGGGTKLELKR

Table S2. Mutual competition of cAbs by enzyme-linked immunosorbent assays

		Indicator cAb								
		T104	T426	AC10	T105	T25	T405	T6	T427	T107
Competitor cAb	T104	11	91	93	94	92	93	95	94	93
	T426	138	13	89	3	145	156	133	119	155
	AC10	107	97	10	101	101	101	22	22	38
	T105	97	46	87	0	95	97	86	91	95
	T25	104	102	101	104	10	95	25	25	76
	T405	106	103	101	108	93	11	106	104	101
	T6	103	102	83	99	88	98	19	20	36
	T427	101	94	85	99	94	97	18	19	36
	T107	104	102	83	99	80	100	28	32	28

Table S3. Mutual competition of cAbs by surface plasmon resonance[a]

		Second antibody, F(ab′)$_2$								
		T104F	T426F	AC10F	T105F	T25F	T405F	T6F	T427F	T107F
	T104		x	x	x	x	x	x	x	x
	T426	x		x		x	x	x	x	x
	AC10	x	x		x	x	x	x		x
First	T105	x		x		x	x	x	x	x
antibody,	T25	x	x	x	x		x	x	x	x
cAb	T405	x	x	x	x	x		x	x	x
	T6	x	x	x	x	x	x		x	x
	T427	x	x		x		x	x		-
	T107	x	x		x	x	x	x		

[a] x and - represent no competition and partial competition, respectively.

Table S4. Sequences of the orthologs and mutants used for epitope determination (separate file)

Table S5. Epitopes of the antibodies determined by mutant analysis

Antibody	Epitope-determining mutants	Precise Location in CD30	Sequences critical for the epitope structure	Previously reported epitope location (25,26)
T104	L1A1	28-34	TCHGNPS	19-68
T426	L3C1	139-144	FPGTAQ	107-153
AC10	L3B1	126-127	FF	19-153
T105	L3C	139-151	FPGTAQKNTVCEP	107-153
T25	MC6AB	288-305	ICATSATNSCARCVPYPI	282-338
T405	M6e	321-335	DTTFEAPPLGTQPDC	282-338
T6	MC5A1	70-76, 245-251 (duplicate)	EPDYYLD	66-107, 224-383 (duplicate)
T427	V2356-SRD, V2356-SAV	88-90 + 117-120, 263-265 + 292-295 (duplicate)	SRD + SAV	19-153, 224-383 (duplicate)
T107	MC5B1	88-89, 263-264 (duplicate)	SR	19-153, 224-383 (duplicate)

Table S6. Production yields of Int^N- or Int^C-fused antibody fragments

	mg/L culture
T104N	52
T426N	86
AC10N	172
T105N	105
T25N	40
T405N	21
T6N	6
T427N	32
T107N	107
T104C	328
T426C	366
AC10C	305
T105C	397
T25C	165
T405C	393
T6C	297
T427C	242
T107C	367

Table S7. Kinetic parameters of antibody interaction with CD30 determined by surface plasmon resonance in a rough-level analysis

Name	k_{on} ($\times 10^{6}$ $M^{-1}s^{-1}$)	k_{off} ($\times 10^{-3}$ s^{-1})	K_D (nM)	Name	k_{on} ($\times 10^{6}$ $M^{-1}s^{-1}$)	k_{off} ($\times 10^{-3}$ s^{-1})	K_D (nM)
T104	0.32	2.8	8.8	BpT25-405	0.29	0.26	0.91
T426	N.D.	N.D.	N.D.	BpT107-6	0.73	0.27	0.37
AC10	1.2	1.6	1.3	BpT107-427	1.0	0.61	0.60
T105	0.70	1.5	2.2	BpT427-6	1.1	0.29	0.26
T25	0.17	1.3	7.5	BpT104-6	1.0	0.61	0.59
T405	0.18	2.4	13	BpT104-427	1.1	0.36	0.32
T6	1.6	1.5	0.94	BpT107-104	1.2	0.47	0.40
T427	1.2	0.64	0.55	BpT10-6	0.98	0.26	0.27
T107	0.10	0.61	6.0	BpT10-427	1.1	0.63	0.58
BpT104-426	1.3	3.9	2.9	BpT10-107	1.1	0.69	0.61
BpT104-25	3.1	1.6	0.50	BpT107-25	0.23	0.20	0.87
BpT104-405	0.16	7.6	48	BpT107-405	0.18	0.33	1.8
BpT426-25	0.37	0.31	0.82	BpT426-6	1.1	0.50	0.45
BpT426-405	0.30	0.61	2.1	BpT426-427	1.2	0.53	0.44
BpT10-104	1.0	0.26	0.26	BpT25-6	0.83	0.39	0.46
BpT10-426	1.1	0.25	0.23	BpT25-427	0.91	0.52	0.57
BpT10-105	0.98	0.065	0.066	BpT105-6	1.1	0.39	0.37
BpT10-25	0.92	0.58	0.64	BpT105-427	0.94	0.23	0.24
BpT10-405	0.98	0.62	0.63	BpT105-107	1.1	0.30	0.26
BpT105-104	0.75	0.24	0.32	BpT405-6	1.1	0.49	0.44
BpT105-426	0.49	1.2	2.3	BpT427-405	1.0	0.32	0.31
BpT105-405	1.4	0.94	0.67	BpT107-426	0.65	0.22	0.34
BpT25-105	1.2	0.65	0.55				

A

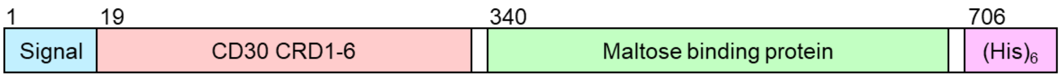

MRVLLAALGLLFLGALRAFPQDRPFEDTCHGNPSHYYDKAVRRCCYRCPMGLFPTQQCPQRPTDCRKQCEPDYYLDEADRC
TACVTCSRDDLVEKTPCAWNSSRVCECRPGMFCSTSAVNSCARCFFHSVCPAGMIVKFPGTAQKNTVCEPASPGVSPACAS
PENCKEPSSGTIPQAKPTPVSPATSSASTMPVRGGTRLAQEAASKLTRAPDSPSSVGRPSSDPGLSPTQPCPEGSGDCRKQ
CEPDYYLDEAGRCTACVSCSRDDLVEKTPCAWNSSRTCECRPGMICATSATNSCARCVPYPICAAETVTKPQDMAEKDTTF
EAPPLGTQPDCNGGSKIEEGKLVIWINGDKGYNGLAEVGKKFEKDTGIKVTVEHPDKLEEKFPQVAATGDGPDIIFWAHDR
FGGYAQSGLLAEITPDKAFQDKLYPFTWDAVRYNGKLIAYPIAVEALSLIYNKDLLPNPPKTWEEIPALDKELKAKGKSAL
MFNLQEPYFTWPLIAADGGYAFKYENGKYDIKDVGVDNAGAKAGLTFLVDLIKNKHMNADTDYSIAEAAFNKGETAMTING
PWAWSNIDTSKVNYGVTVLPTFKGQPSKPFVGVLSAGINAASPNKELAKEFLENYLLTDEGLEAVNKDKPLGAVALKSYEE
ELVKDPRIAATMENAQKGEIMPNIPQMSAFWYAVRTAVINAASGRQTVDEALKDAQGHHHHHH

B **C**

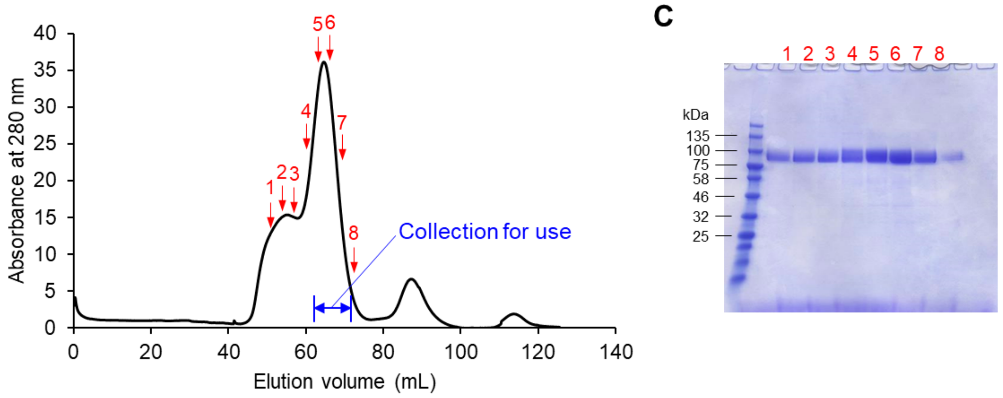

Fig. S1. Maltose-binding protein-fused CD30 (CD30-MBP). A) Construction of CD30-MBP and the peptide sequence. B) Size-exclusion chromatogram for the final step of CD30-MBP purification. C) SDS-PAGE analysis of peak fractions from size-exclusion chromatography. Red-numbered arrows in B correspond to lanes in C.

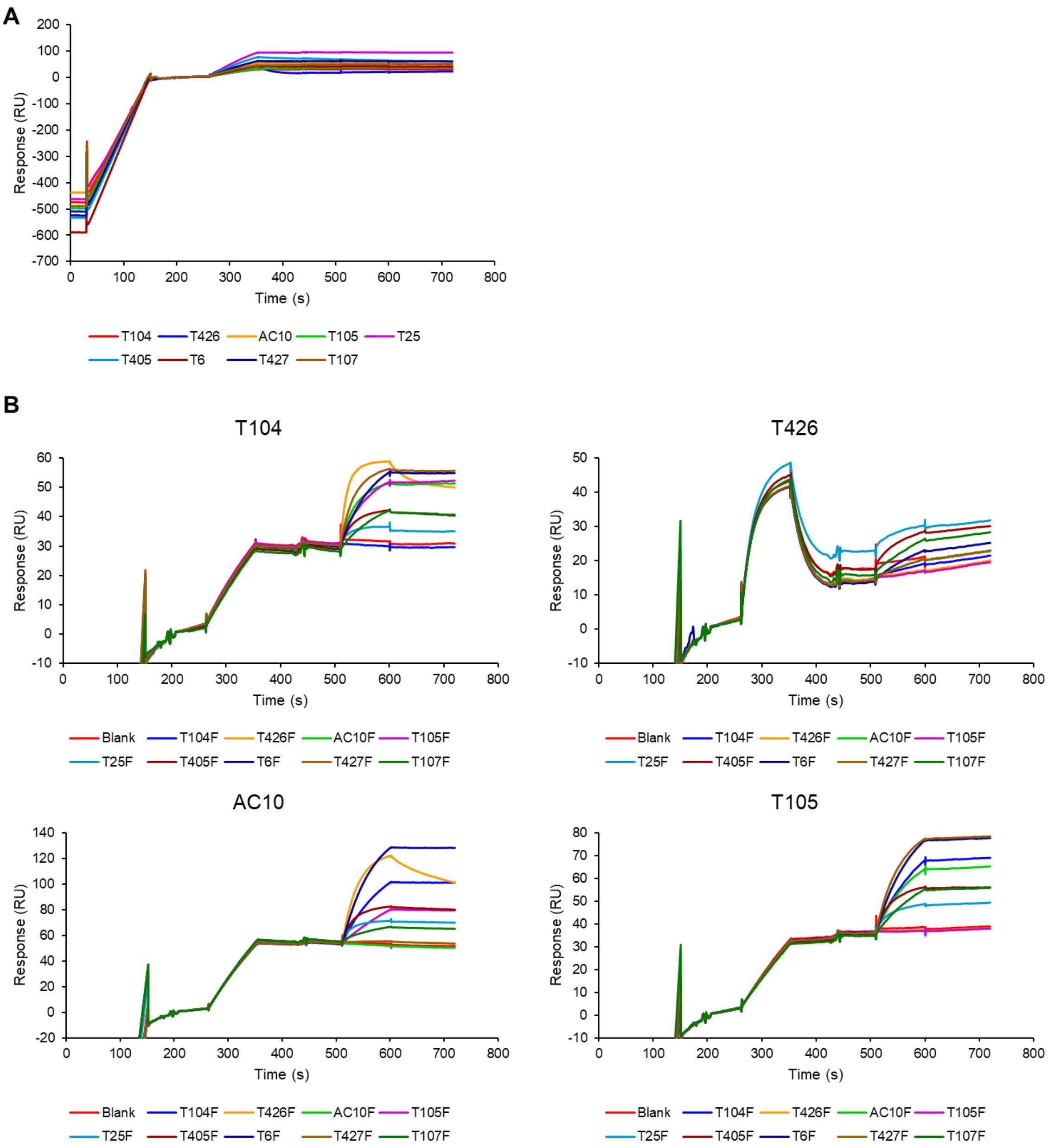
A
Response (RU)
Time (s)
T104
T426
AC10
T105
T25
T405
T6
T427
T107
B
T104
T426
AC10
T105
Response (RU)
Time (s)
Blank
T104F
T426F
AC10F
T105F
T25F
T405F
T6F
T427F
T107F

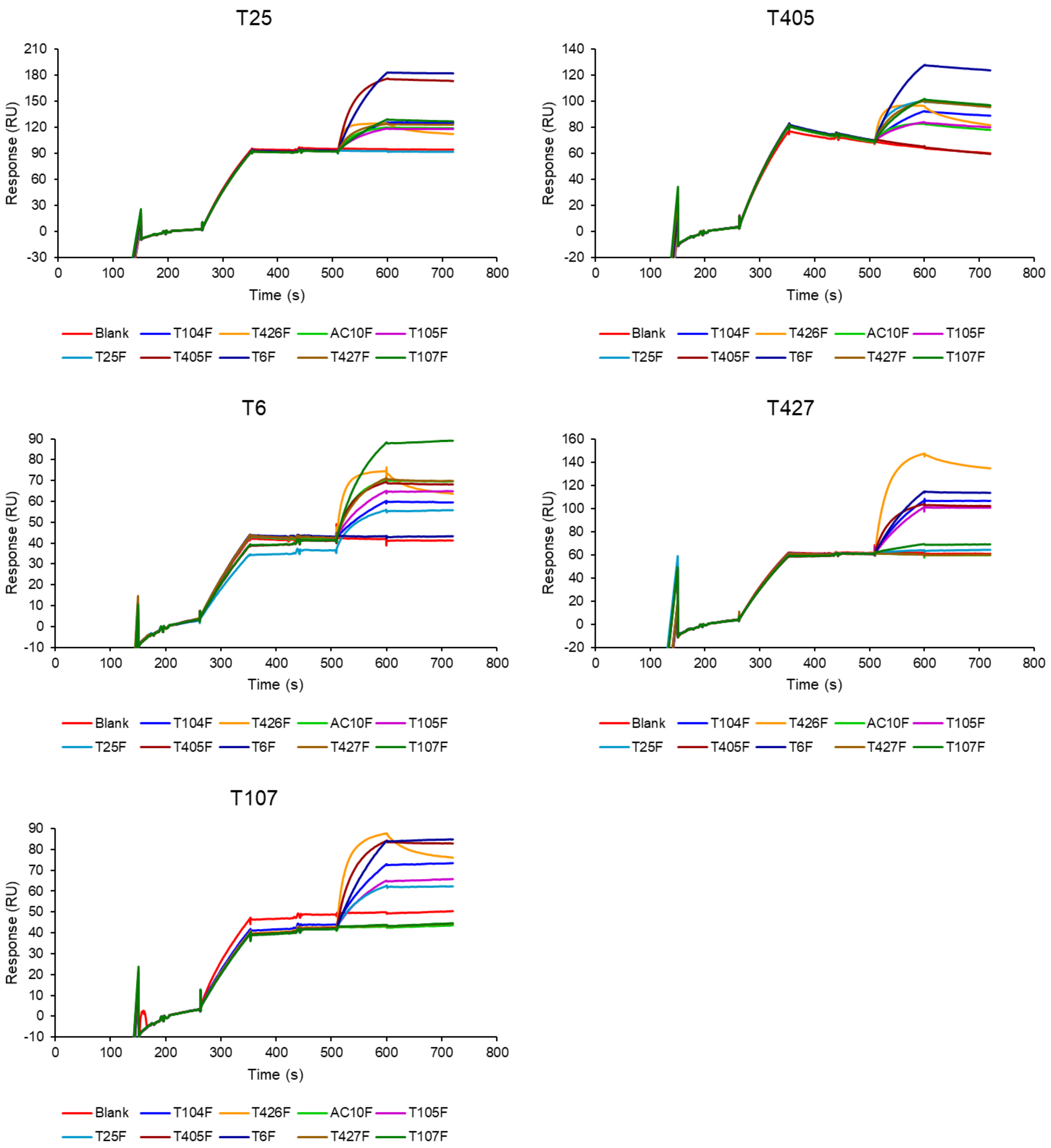

Fig. S2. Surface plasmon resonance sensorgrams for evaluating competitive binding by variable regions. A) Capture of cAbs by anti-Fc (30–150 s), followed by capture of CD30-MBP by the cAbs (270–350 s). These runs correspond to the ‘Blank’ runs in B. B) Interaction of F(ab′)$_2$ antibodies with CD30-MBP captured by the indicated cAbs on top of the panels. To capture CD30-MBP as described above, F(ab′)$_2$ antibodies were flowed to contact (510–600 s), followed by dissociation.

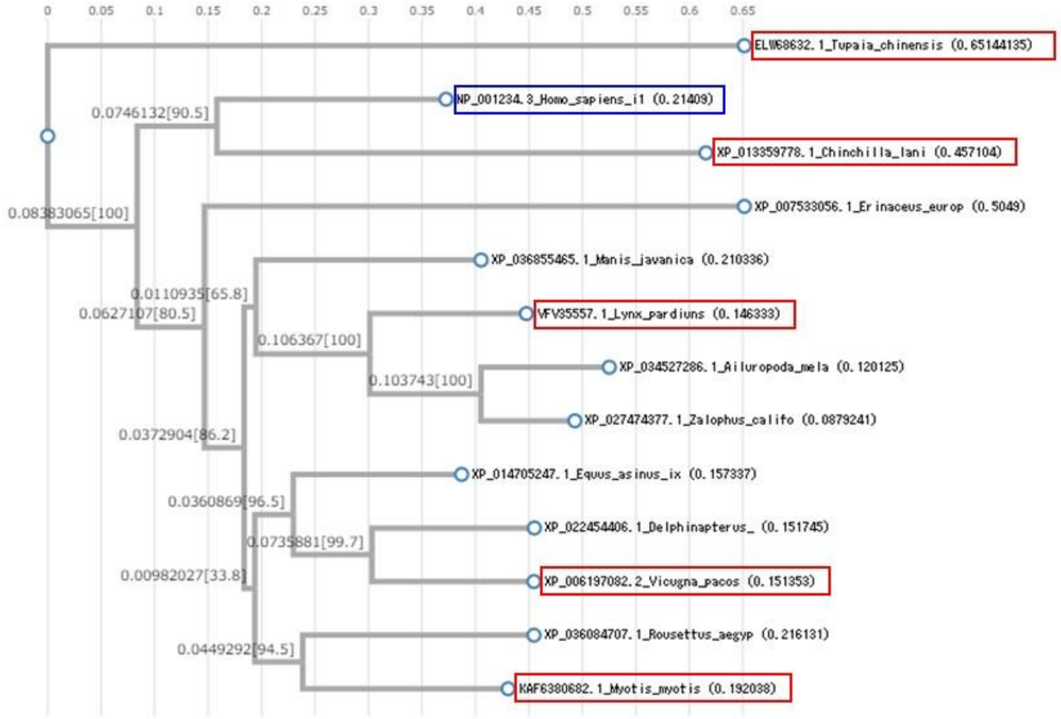

Fig. S3. Phylogenetic tree of human CD30 proteins (blue) and five orthologs (red) among selected sequences.

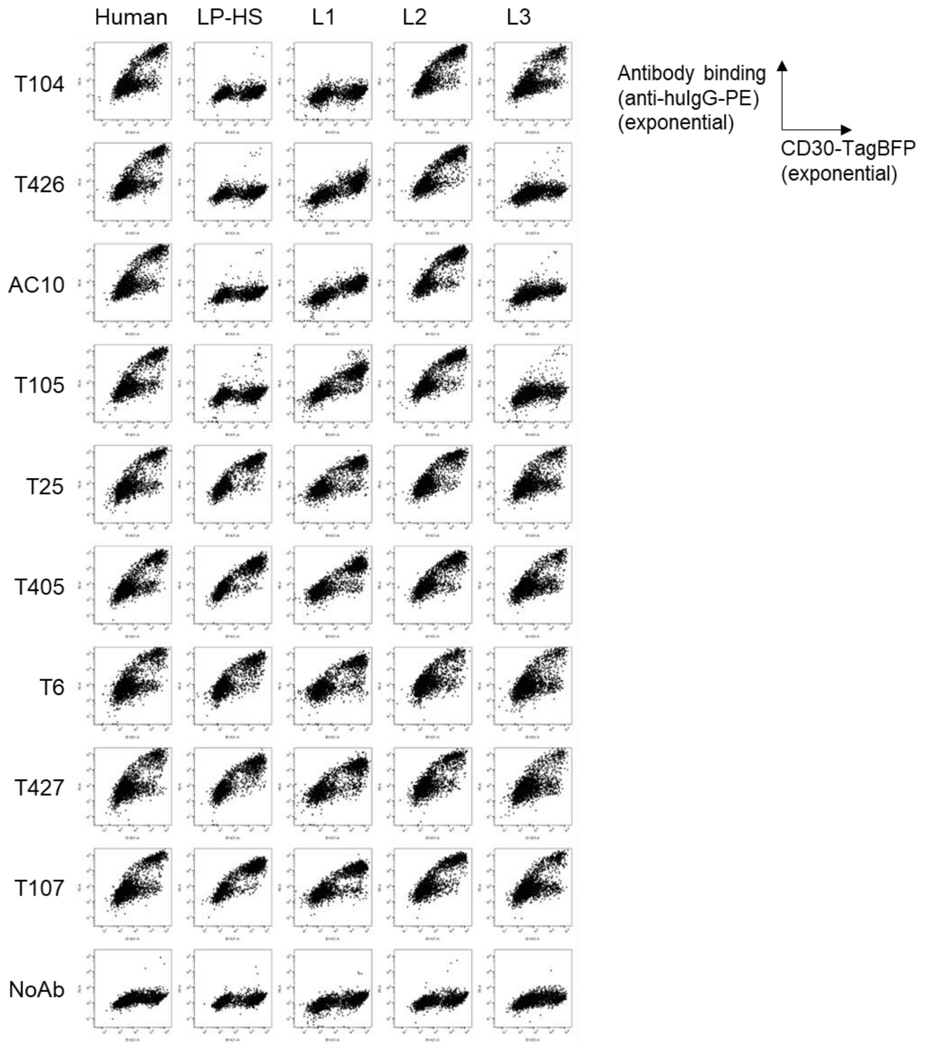

Fig. S4. Antibody binding to human CD30 and domain-substituted CD30 proteins with L.pard sequences in CRD1-3 (LP-HS), CRD1 (L1), CRD2 (L2), and CRD3 (L3).

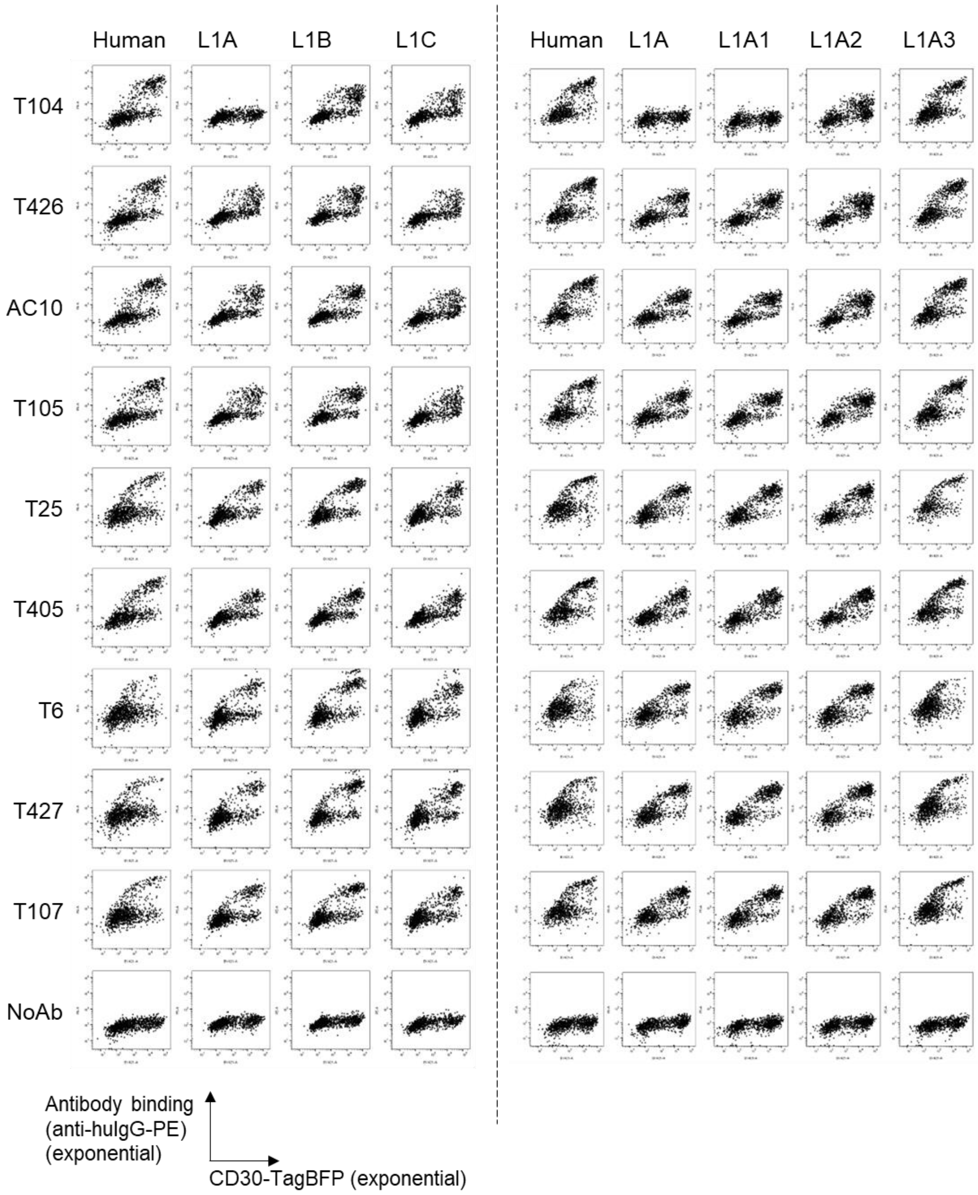

Fig. S5. Antibody binding to human CD30 with a portion of CRD1 substituted with L.pard sequences. Split line indicates data from independent experiments.

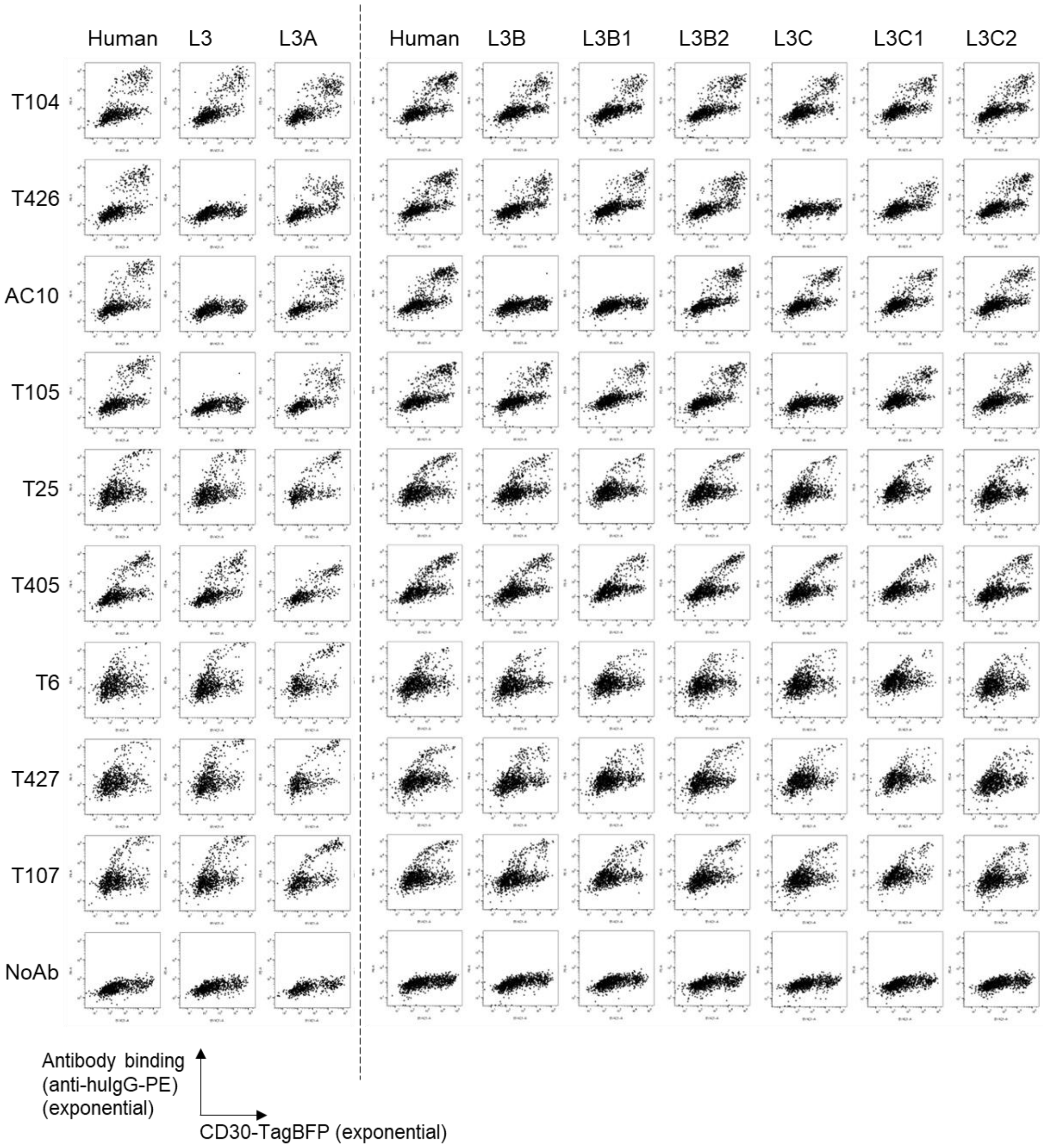

Fig. S6. Antibody binding to human CD30 with a portion of CRD3 substituted with L.pard sequences. Split line indicates data from independent experiments.

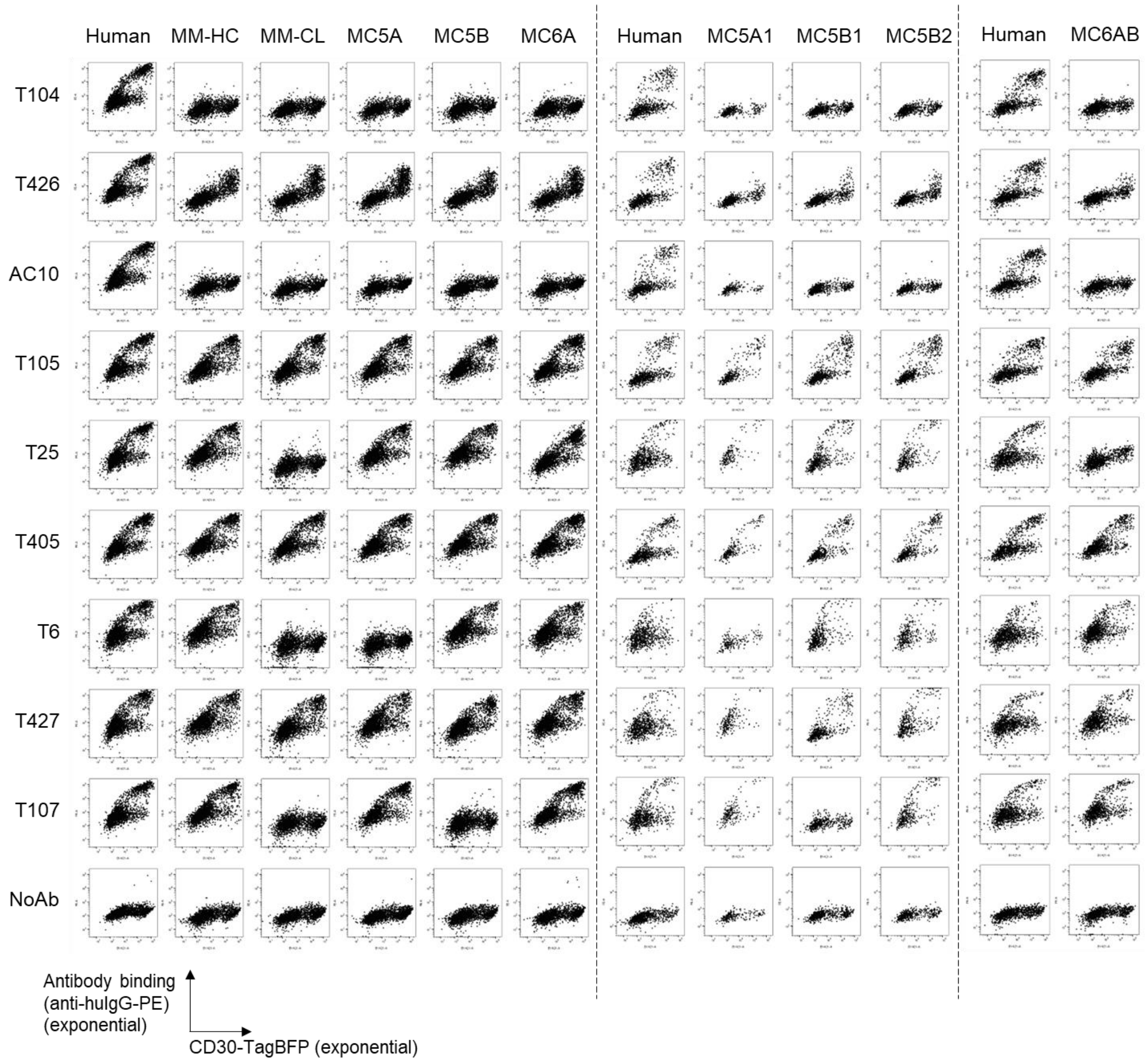

Fig. S7. Antibody binding to human CD30 with entire CRD1-3 substituted with M.myot sequence and a portion of CRD1 substituted with C.lani sequences. Split line indicates data from independent experiments.

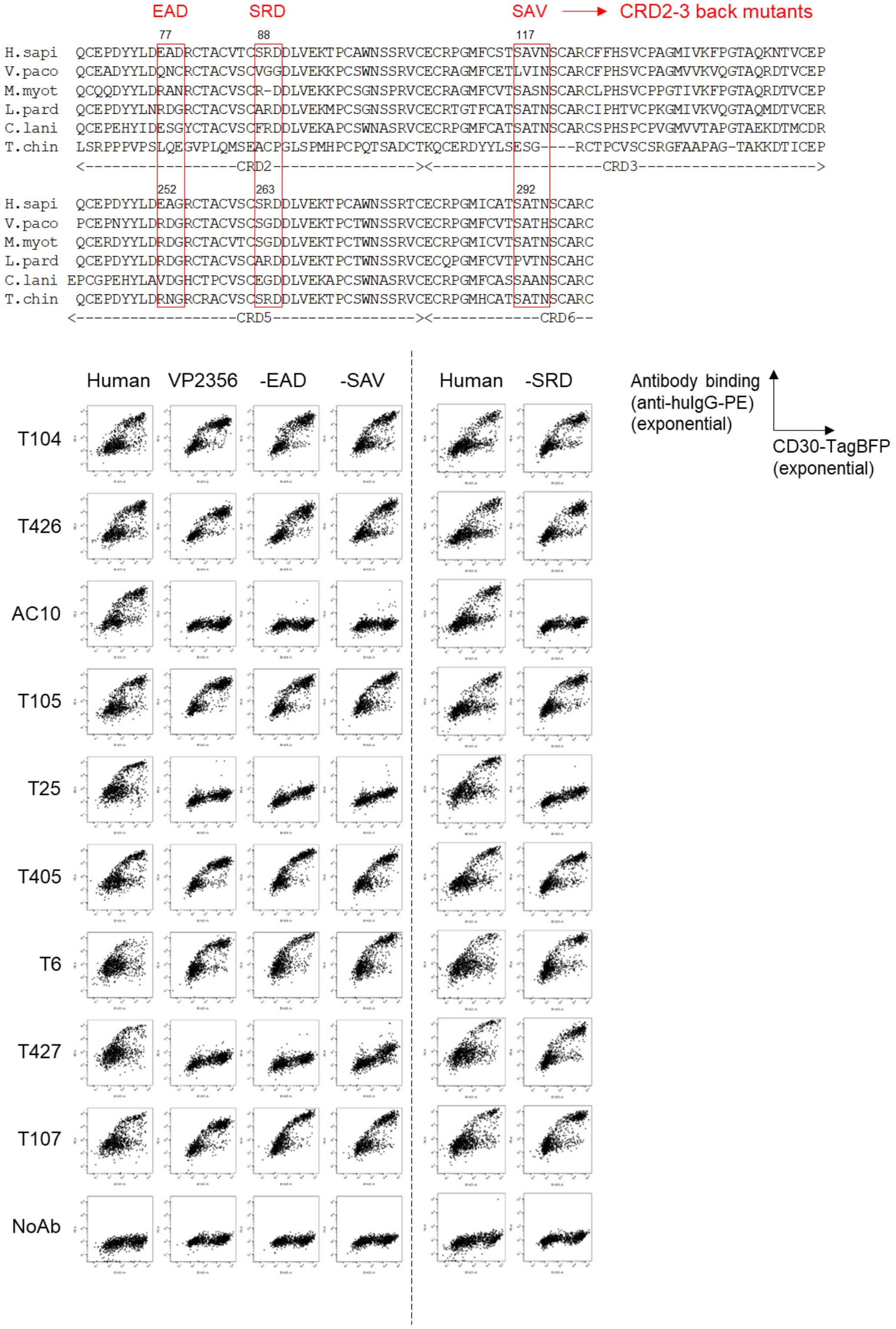

Fig. S8. Design of V.paco mutants and antibody binding to human CD30 with CRD2-3 and CRD5-6 substituted with V.paco sequences. "-EAD," "-SAV," and "-SRD" indicate back mutations to human sequences. Split line indicates data from independent experiments.

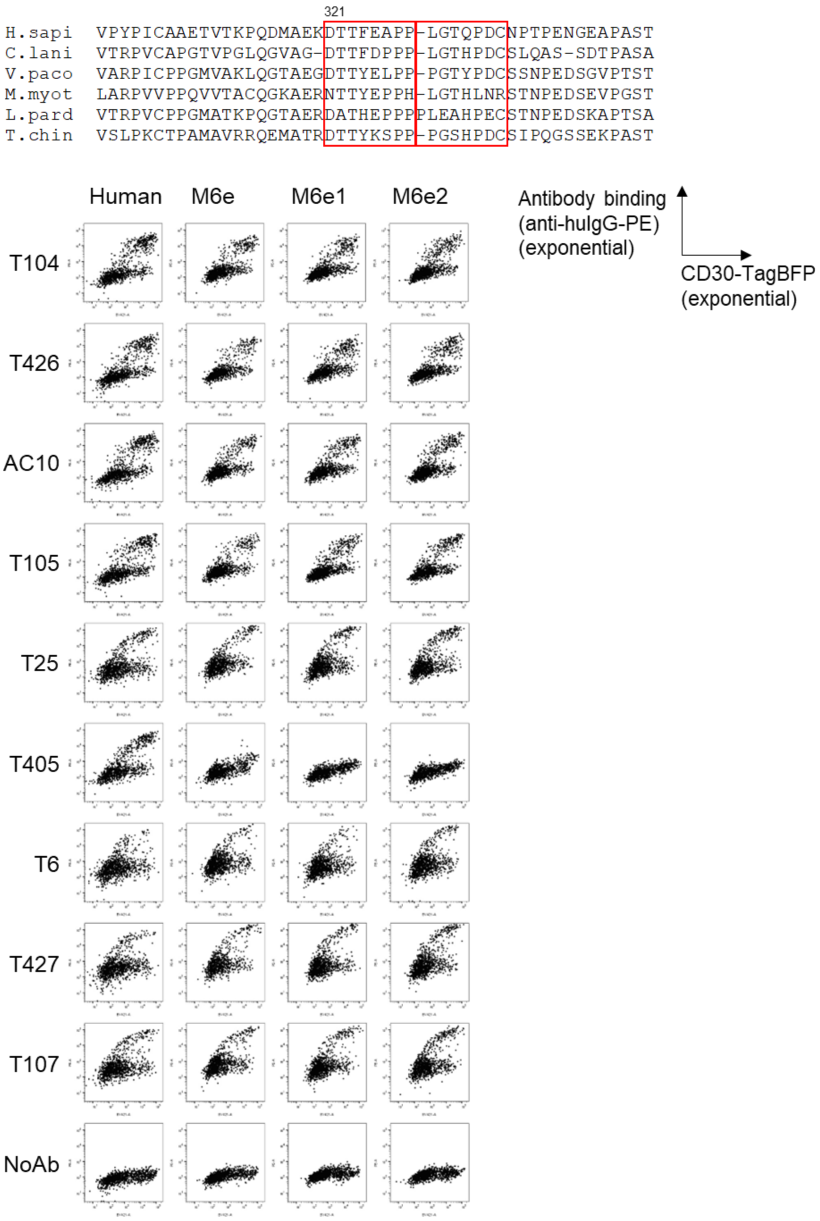

Fig. S9. Design of M.myot mutants and antibody binding to human CD30 with a portion of CRD6 or its C-terminal region substituted with M.myot sequences.

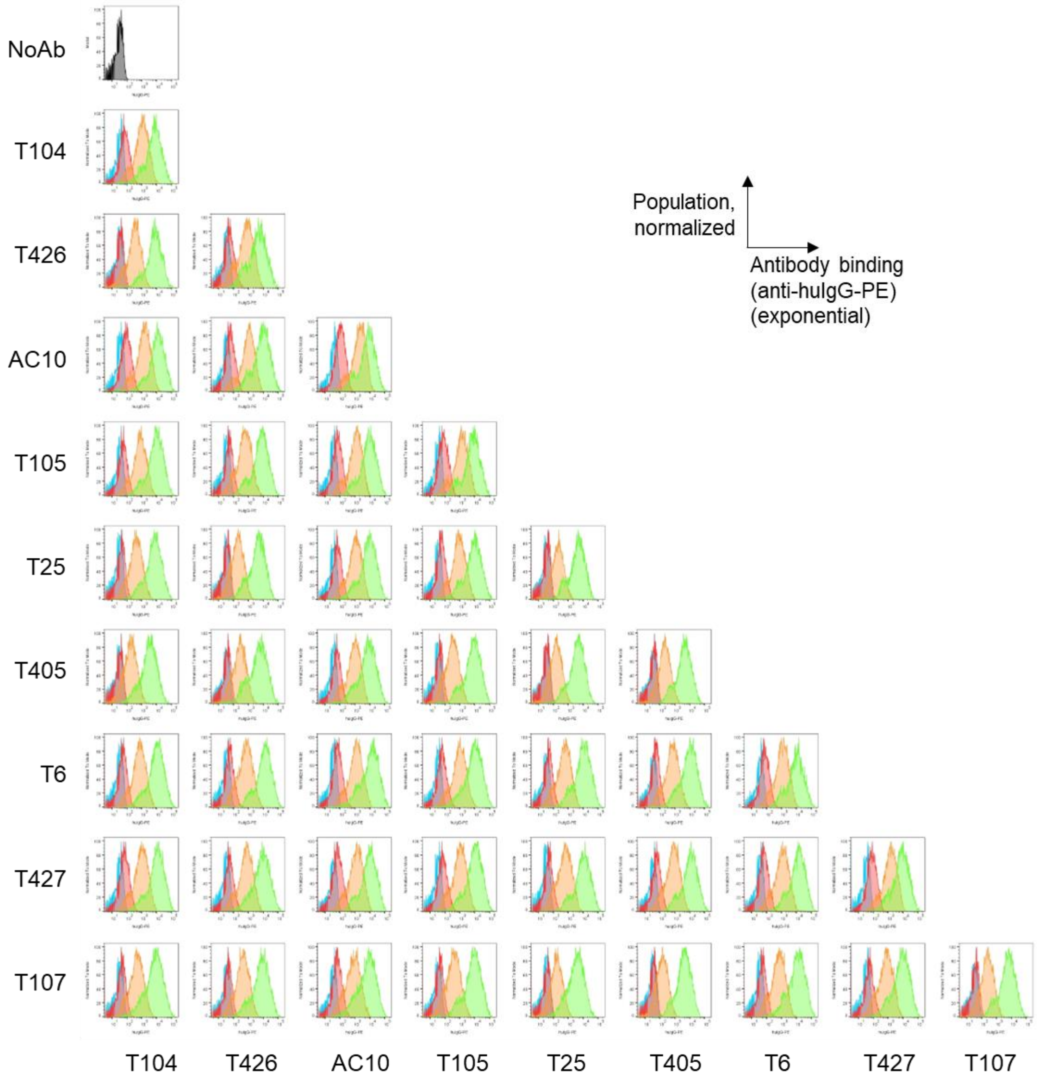

Fig. S10. Antibody binding to CD30-transfected Ramos-blue cells at various concentrations. Antibodies were tested at concentrations of 1500 ng/mL (green), 15 ng/mL (orange), 0.15 ng/mL (red), or 0.0015 ng/mL (cyan). Each panel corresponds to a specific antibody configuration: when x- and y-axis labels are the same (e.g., both are T104), it represents the binding of cAb (e.g., T104). Conversely, when x- and y-axis labels are different (e.g., x-axis label is T104 and y-axis label is T426), it signifies the binding of BpAb with the labeled Fvs (e.g., BpT104-426).

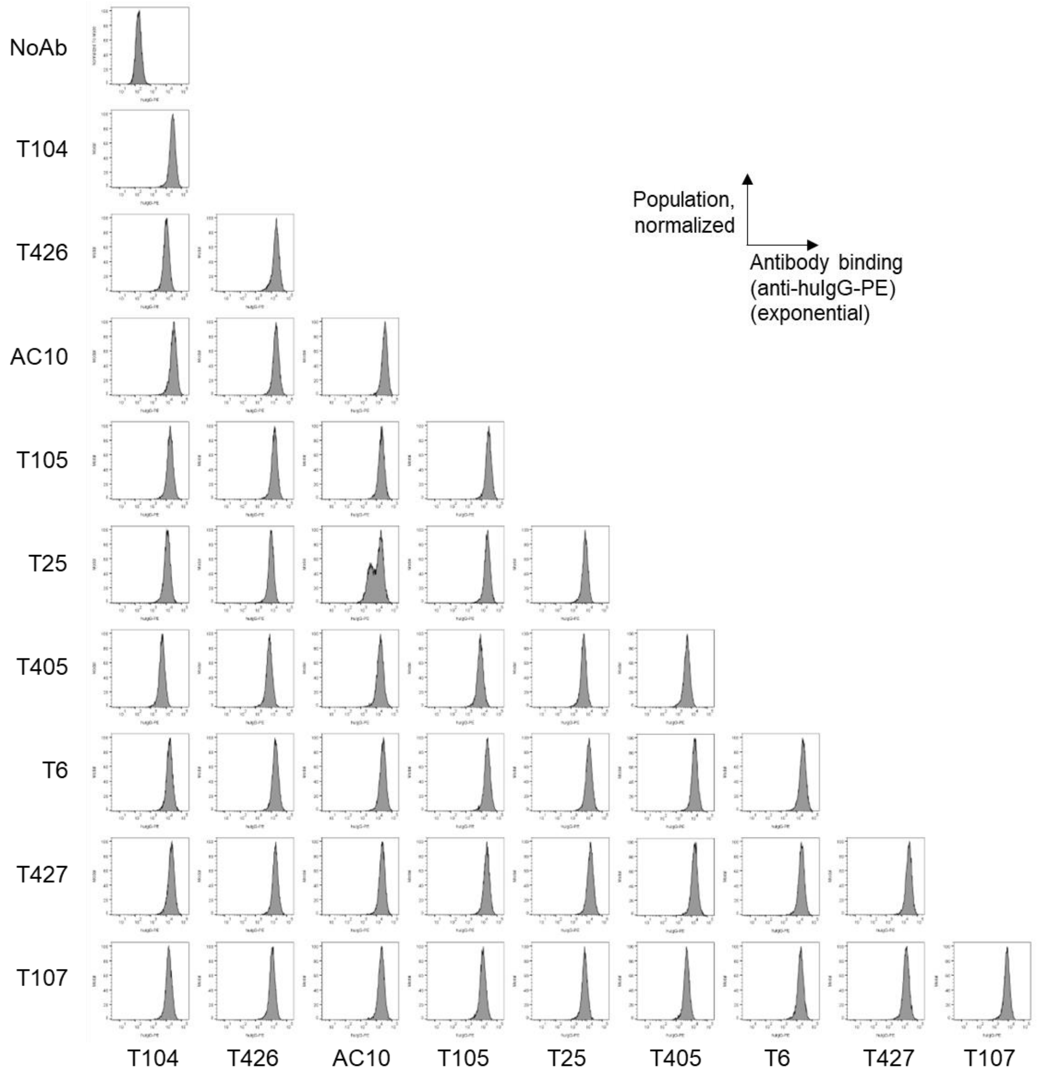

Fig. S11. Antibody binding to KARPAS 299 cells at 150 ng/mL. For each panel, when x- and y-axis labels are the same (e.g., both are T104), the panel corresponds to binding of cAb (e.g., T104). When x- and y-axis labels are different (e.g., x-axis label is T104 and y-axis label is T426), the panel corresponds to binding of BpAb with labeled Fvs (e.g., BpT104-426).

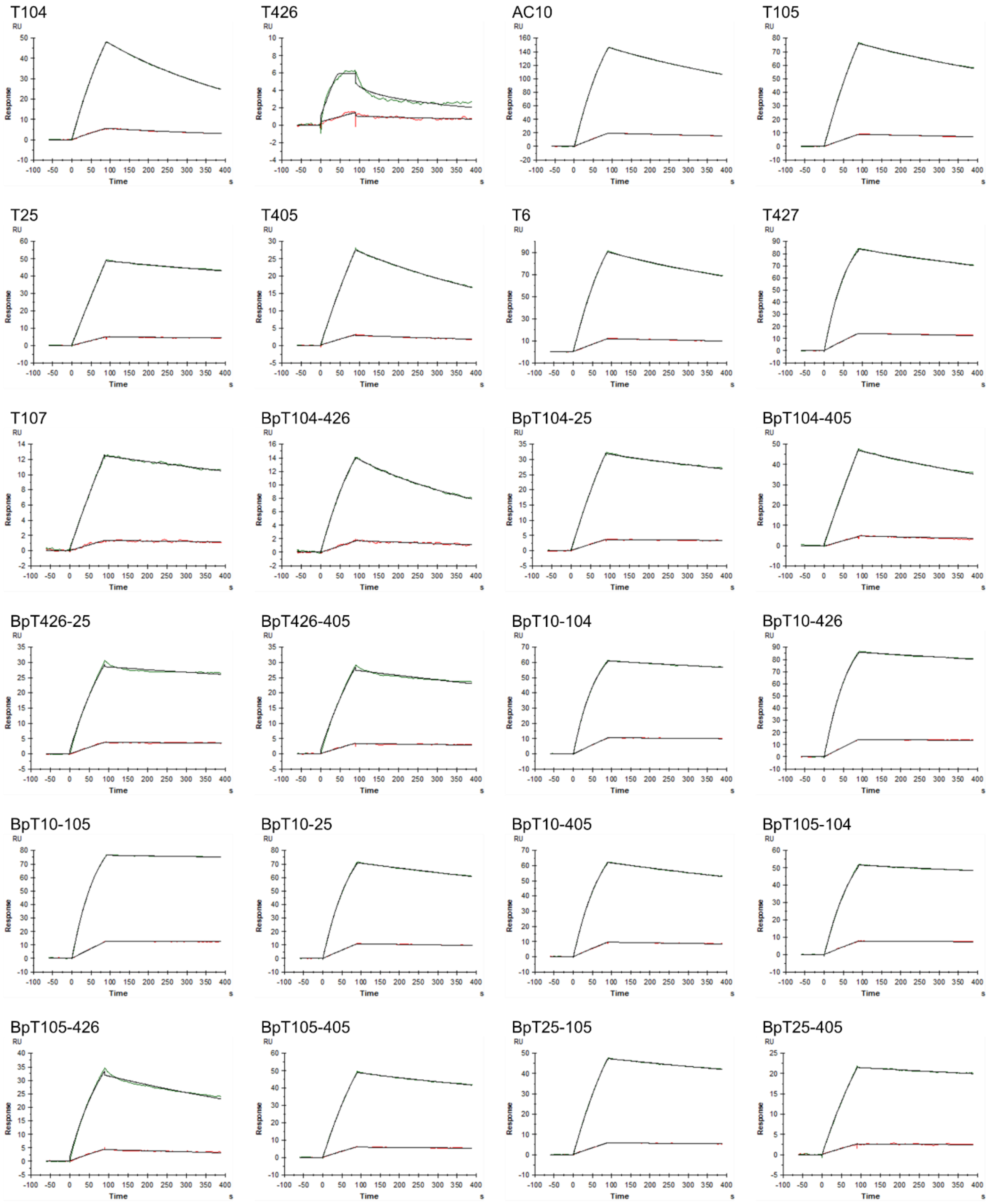
T104
T426
AC10
T105
T25
T405
T6
T427
T107
BpT104-426
BpT104-25
BpT104-405
BpT426-25
BpT426-405
BpT10-104
BpT10-426
BpT10-105
BpT10-25
BpT10-405
BpT105-104
BpT105-426
BpT105-405
BpT25-105
BpT25-405
RU
Response
Time
s

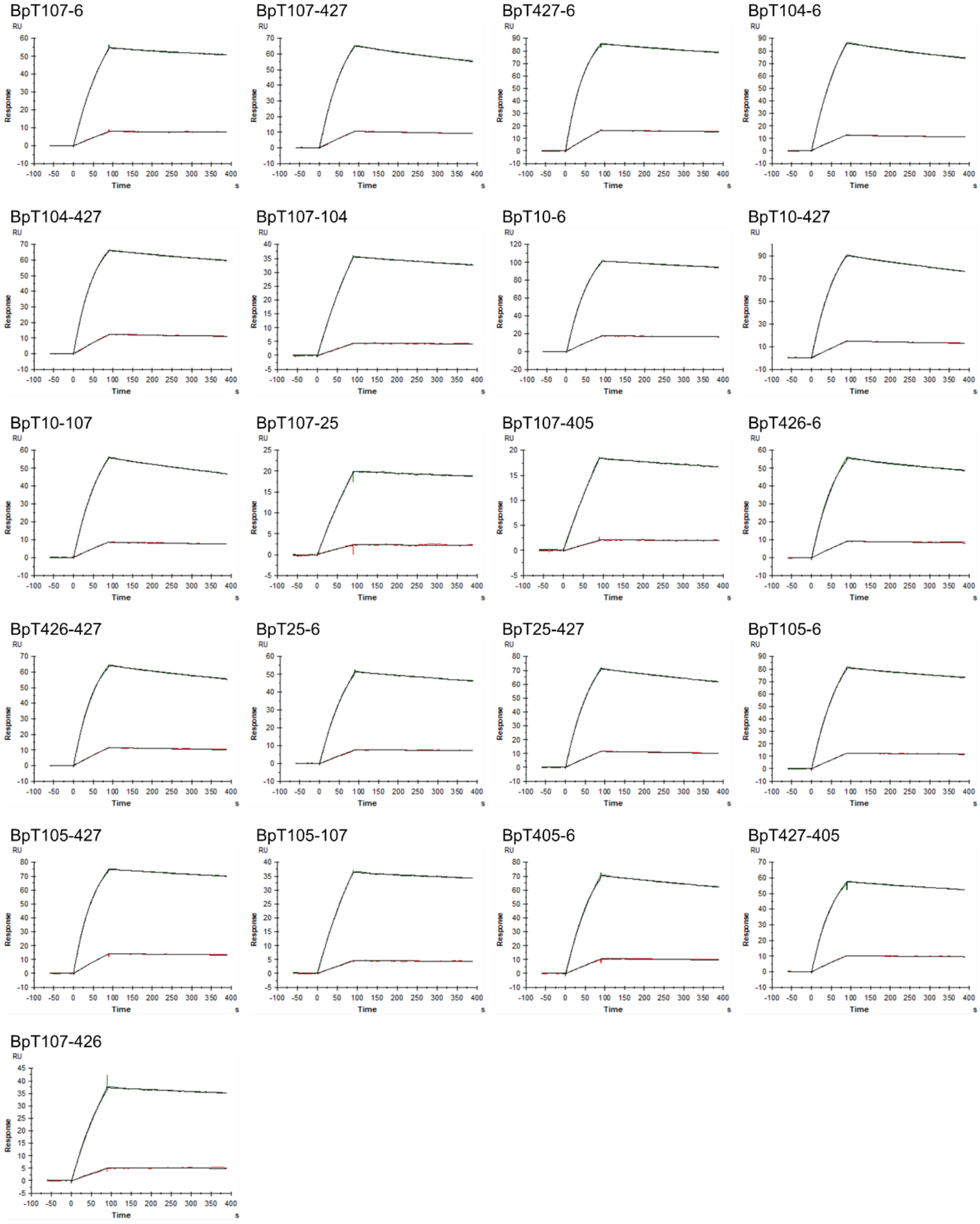

Fig. S12. Surface plasmon resonance charts of CD30-MBP binding to immobilized antibodies. CD30-MBP was flowed at concentrations of 2 nM (red) or 20 nM (green) for all antibodies except T426. The sensorgrams are displayed with the baseline subtracted for CD30-MBP flowed at 0 nM. For T426, the concentrations were 20 nM (red) or 200 nM (green). Black lines represent the fitting curves for a 1:1 binding mode.

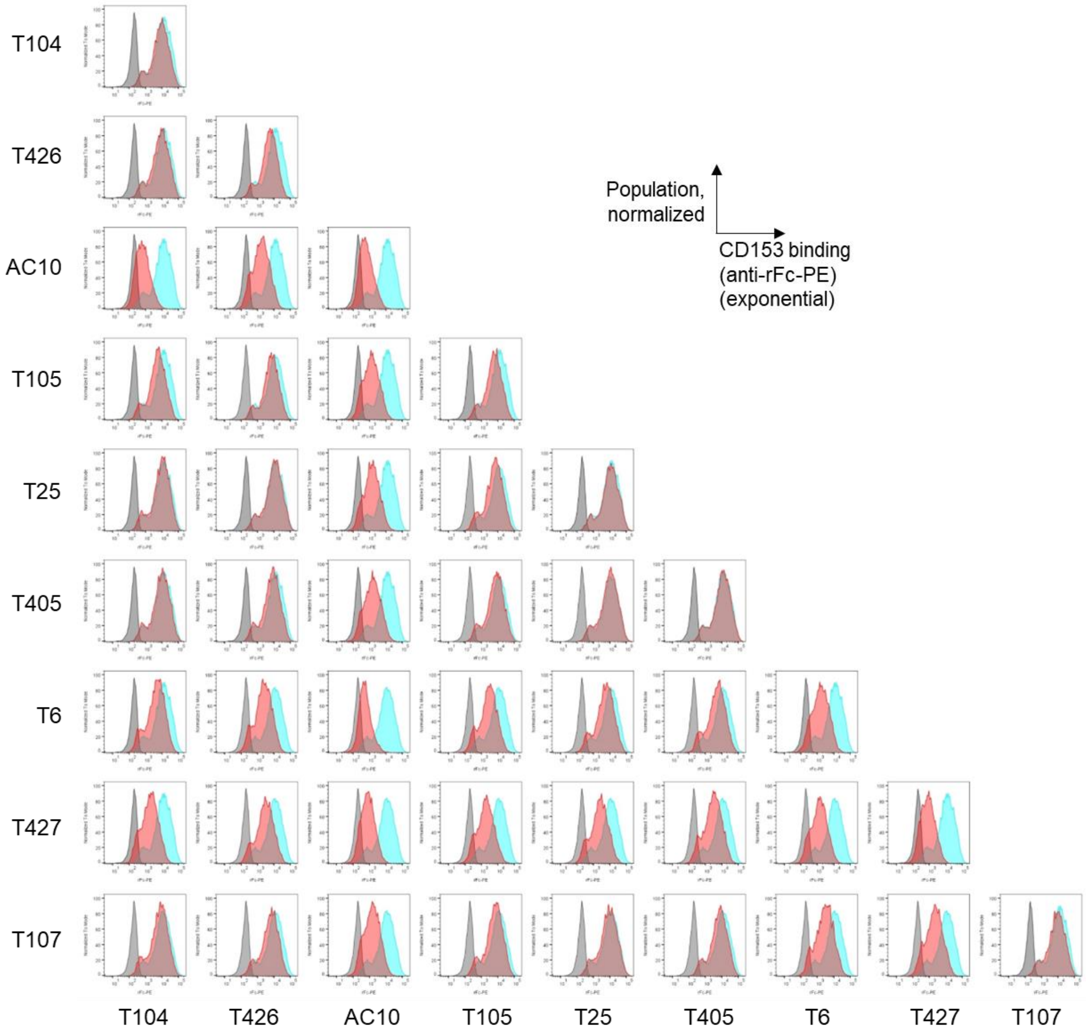

Fig. S13. Binding inhibition of CD153-rFc by cAbs and BpAbs. The binding of CD153-rFc to CD30-RamosBlue cells was assessed using flow cytometry. In the graphs, grey represents the secondary antibody (anti-rabbit Fc-PE) only, cyan indicates CD153-rFc alone, and red shows CD153-rFc in the presence of the antibody. Each panel corresponds to either cAb competition (e.g., T104) or BpAb competition with labeled Fvs (e.g., BpT104-426), depending on the alignment of the x- and y-axis labels.

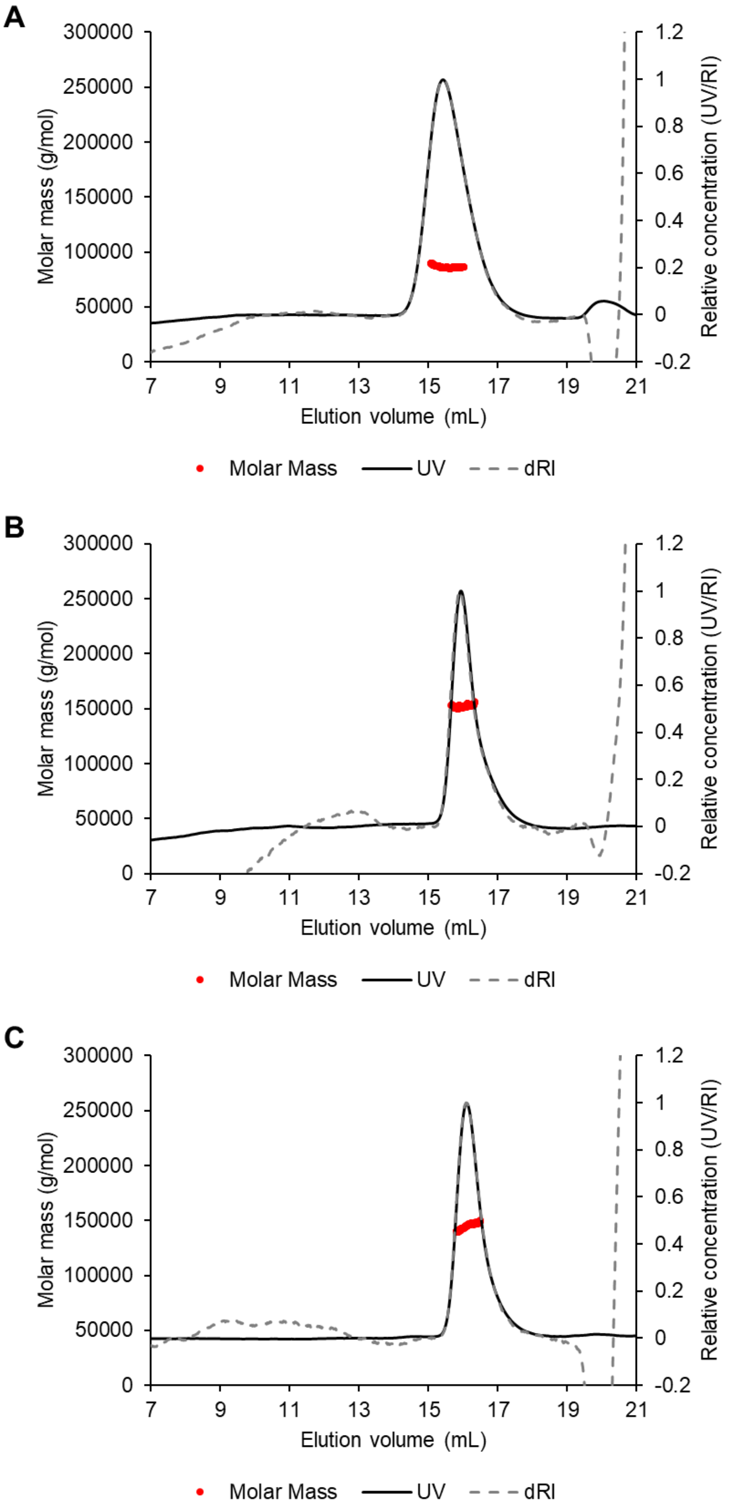

Fig. S14. SEC-MALS charts for individual components. Panels display the results for CD30-MBP alone (A), BpT10-104 alone (B), and BpT10-405 alone (C), each analyzed separately outside of the complex.
